# Supplementary material for: Structure and function relationship of OqxB efflux pump from Klebsiella pneumoniae
Source: Nat Commun. 2021 Sep 13;12:5400. doi: 10.1038/s41467-021-25679-0 (PMC8437966; doi:10.1038/s41467-021-25679-0)
Supplement: Supplementary file 1 — Supplementary information [file 41467_2021_25679_MOESM1_ESM.pdf]

Structure and Function relationship of OqxB efflux pump from *Klebsiella pneumoniae*

Nagakumar Bharatham[1,2], Purnendu Bhowmik[1,2], Maho Aoki[3], Ui Okada[3], Sreevalli Sharma[1,2], Eiki Yamashita[4], Anirudh P. Shanbhag[1], Sreenath Rajagopal[1], Teby Thomas[5], Maitrayee Sarma[1], Riya Narjari[1], Savitha Nagraj[6], Vasanthi Ramachandran[1,2], Nainesh Katagihallimath[1,2], Santanu Datta[1], Satoshi Murakami[3*]

[1]Bugworks Research India Pvt. Ltd., Centre for Cellular and Molecular Platforms, GKVK, Bellary Rd, Bengaluru, Karnataka-560065, India.

[2]The University of Trans-Disciplinary Health Sciences and Technology (TDU), Bengaluru, Karnataka-560064, India.

[3]Department of Life Science & Technology, Tokyo Institute of Technology, 4259 Nagatsuta, Midori-ku, Yokohama 226-8501, Japan.

[4]Institute for Protein Research, Osaka University, Suita, Osaka, 565-0871, Japan.

[5]St. John's Research Institute, Bengaluru, Karnataka-560034, India.

[6]St. John's Medical Hospital, Bengaluru, Karnataka-560034, India.

***Corresponding Author**: Prof. Satoshi Murakami

E. Mail: murakami@bio.titech.ac.jp

Department of Life Science & Technology, Tokyo Institute of Technology, 4259 Nagatsuta, Midori-ku, Yokohama 226-8501, Japan.

Supplementary information

Supplementary Table 1: Crystallographic data and refinement statistics

**Data collection**	***K. pneumoniae* OqxB**
Space group	*P*1
Cell dimensions	
a, *b*, *c* (Å)	128.9, 128.8, 137.3
α, β, γ (°)	91.3, 90.0, 103.6
Wavelength (Å)	0.9000
Resolution (Å)	1.85 (1.88 – 1.85)
No. reflections	
Observed	2,708,687 (133,968)
Unique	709,740 (34,205)
Redundancy	3.8 (3.9)
R_{merge}	0.073 (>1.0)
$CC_{1/2}$	1.00 (0.48)
Completeness (%)	96.7 (93.2)
$I/\sigma(I)$	8.9 (0.9)
**Refinement**	
Resolution (Å)	43.28 – 1.85
R / R_{free}	0.1802 / 0.2169
rms deviation from ideal	
Bond lengths (Å)	0.018
Bond angles (˚)	1.408
Average *B* factors (Å^2)	47.80 (protein), 49.78 (overall)
Ramachandran plot (%)	
Favored	97.64
Allowed	2.10
Disallowed	0.26

Values in parentheses are for the highest resolution shell

Supplementary Table 2: Structural similarity of individual protomers.

	A	B	C	D	E	F
**A**	---	**0.589**	**0.659**	**0.592**	**0.281**	**0.528**
**B**		---	**0.621**	**0.734**	**0.546**	**0.553**
**C**			---	**0.557**	**0.611**	**0.675**
**D**				---	**0.601**	**0.737**
**E**					---	**0.490**
**F**						---

Pairwise main chain RMSDs calculated by superposition method and values represented in Å. LSQKAB in CCP4 program suite was used for main chain atoms (1-1039).

Supplementary Table 3: Minimum Inhibitory Concentrations (MIC) of ciprofloxacin against *E. coli* clinical isolates and mutation mapping by sequencing

Strain	MIC (µg/mL)	*Ec*GyrA*		*Ec*ParC*			
E. coli	**Ciprofloxacin**	**S83L**	**D87N**	**S80I**	**S80R**	**E84V**	**E84G**
SEC001	>8	+	+	+			
SEC002	>8	+	+	+			
SEC003	>8	+	+	+			
SEC004	>8	+	+	+			
SEC005	>8	+	+				
SEC006	>8	+	+	+			
SEC007	>8	+	+				+
SEC008	>8	+	+	+			
SEC009	>8	+	+	+			
SEC010	0.25	+	+				
SEC011	>8	+	+	+			
SEC012	>8		+	+			
SEC013	>8	+	+	+			
SEC014	>8	+	+	+			
SEC015	>8	+	+				
SEC016	>8	+	+	+			
SEC018	>8	+	+	+			
SEC019	>8	+	+	+			
SEC020	>8		+	+			
SEC021	>8	+		+		+	
SEC022	>8	+	+	+		+	
SEC023	>8	+	+				
SEC024	>8	+	+	+			
SEC025	>8	+	+				
SEC026	>8	+	+	+			
SEC027	>8	+	+				
SEC028	>8	+	+	+		+	
SEC029	>8	+	+	+		+	
SEC030	>8	+	+	+		+	
SEC031	>8	+	+	+		+	
SEC032	0.03						
SEC033	>8	+	+				
SEC034	>8	+	+	+			
SEC035	>8			+			
SEC036	>8	+	+	+			
SEC037	0.015						
SEC038	>8	+	+	+		+	
SEC039	>8	+	+	+			
SEC040	>8	+	+	+			
SEC041	>8	+	+	+			+
SEC042	>8	+	+	+			

SEC043	>8	+	+	+		+	
SEC044	>8	+	+	+			
SEC045	0.06	+					
SEC046	>8	+	+				
SEC047	>8		+	+			
SEC048	>8	+	+	+			
SEC049	>8	+	+	+			
SEC050	>8			+		+	
SEC051	0.25	+	+	+			
SEC052	0.25	+		+			
SEC053	>8	+	+	+			
SEC054	>8	+					
SEC055	>8	+					
SEC056	0.25	+					
SEC057	>8	+	+		+		
SEC058	>8	+	+				
SEC059	>8	+		+			
SEC061	>8	+	+	+			
SEC062	>8		+				
SEC063	0.125						
SEC064	>8	+	+	+			
SEC065	>8	+	+	+		+	
SEC066	>8	+					
SEC067	>8	+	+	+			
SEC068	>8	+	+	+			
SEC069	>8	+	+	+		+	
SEC070	>8	+	+	+			
SEC071	>8			+			
SEC072	>8	+	+	+			
SEC073	>8	+	+	+		+	
SEC074	>8	+	+	+		+	
SEC075	>8	+	+	+			
SEC076	>8	+	+	+		+	
SEC077	>8	+	+	+			+
SEC078	>8	+	+	+			+
SEC079	>8	+	+	+		+	
SEC081	>8	+	+	+			
SEC082	>8	+	+	+			
SEC084	>8	+	+	+			
SEC085	>8	+	+	+		+	
SEC086	0.015						
SEC087	>8	+	+	+			
SEC088	>8	+	+	+		+	

*Ciprofloxacin targets GyrA and ParC were sequenced and variations in key interacting residues were identified. Mutations in QRDR (Quinolone-resistance determining region) are highlighted with + symbol.

Supplementary Table 4: Minimum Inhibitory Concentrations (MIC) of ciprofloxacin with and without AcrB inhibitor PAβN

	**Ciprofloxacin MIC (μg/mL)**		**Linezolid MIC (μg/mL)**		***Ec*GyrA***		***Ec*ParC***			
	**Without PAβN**	**With PAβN**	**Without PAβN**	**With PAβN**	**S83L**	**D87N**	**S80I**	**S80R**	**E84V**	**E84G**
SEC010	0.25	0.25	400	40	+	+				
SEC030	200	100	400	40	+	+	+		+	
SEC032	0.06	0.015	400	40						
SEC037	0.015	0.015	200	20						
SEC045	0.25	0.125	200	20	+					
SEC052	0.25	0.25	200	20	+		+			
SEC056	0.25	0.25	200	10	+					
SEC063	0.25	0.25	400	20						
SEC086	0.008	0.008	200	20						
BW25113	0.015	0.015	200	5						
acrB KO	0.008	0.008	5	5						
*tol*C KO	0.004	0.004	5	5						

* Ciprofloxacin targets GyrA and ParC were sequenced and variations in key interacting residues were identified. Mutations in QRDR (Quinolone-resistance determining region) are highlighted with + symbol. Linezolid is the positive control.

Supplementary Table 5: Profiling of 84 clinical strains by colony PCR method to verify presence of *oqxB*

	Ciprofloxacin MIC (μg/mL)		Linezolid MIC (μg/mL)		*oqxB*	*Ec*GyrA*		*Ec*ParC*			
	Without PAβN	With PAβN	Without PAβN	With PAβN		S83L	D87N	S80I	S80R	E84V	E84G
SEC027	>320	160	320	20	-	+	+				
SEC029	160	80	160	5	-	+	+	+		+	
SEC046	40	40	160	5	-	+	+				
SEC028	160	160	320	10	#	+	+	+		+	
SEC033	80	40	320	40	#	+	+				
SEC058	10	10	160	40	#	+	+				

*Ciprofloxacin targets GyrA and ParC were sequenced and variations in key interacting residues were identified. The presence of *oqxB* in the clinical isolates is highlighted with # symbol. Mutations in QRDR (Quinolone-resistance determining region) are highlighted with + symbol. Linezolid is the positive control.

Supplementary Table 6: Sequence identity & similarity between OqxB and other RND pumps

RND pump	Identity	Strongly similar	Weakly similar	Different
OqxB vs AcrB	40.34%	25.26%	11.4%	23%
OqxB vs MexB	41.68%	23.63%	12.29%	22.4%
AcrB vs MexB	69.71%	15.05%	6.76%	8.48%

ClustalW sequence alignment method utilized to calculate the identity metrics.

Supplementary Table 7: Probable OqxB erythromycin binding pocket residues comparison with other RND pumps

	MtrD (PDB ID: 6VKT)	AcrB (PDB ID: 4DX5)	MexB (PDB ID: 6IIA)	OqxB (PDB ID: 7CZ9)
**Erythromycin binding pocket residues**	F136	F136	F136	L138
	I139	V139	V139	V141
	M141	G141	G141	H143
	R174	Q176	Q176	Q178
	F176	F178	F178	F180
	S275	I277	I277	L280
	T277	A279	A279	S282
	G286	G288	G288	G291
	Y325	Y327	Y327	Y330
	F568	V571	V571	Y579
	V607	V612	V612	F618
	F610	F615	F615	L621
	F612	F617	F617	A623
	F623	F628	F628	F636
	----	----	----	F626

Supplementary Table 8: Distance measurement between PC1 and PC2 sub-domain residues to differentiate cleft opening and closing.

PDB_chainID	Organism	RND Pump	Protomer type*	Distance1 (D1)	Distance 2 (D2)	Distance 3 (D3)
4DX5_A	*E. coli*	AcrB	A/L	12.5	15	17.5
4DX5_B	*E. coli*	AcrB	B/T	15.2	17.2	18.5
4DX5_C	*E. coli*	AcrB	E/O	8.57	9.24	12.3
4DX6_A	*E. coli*	AcrB	A/L	12.7	14.1	16.7
4DX6_B	*E. coli*	AcrB	B/T	14.5	15.8	17.1
4DX6_C	*E. coli*	AcrB	E/O	9.2	9.2	11.8
4DX7_A	*E. coli*	AcrB	A/L	12.2	14.2	17.1
4DX7_B	*E. coli*	AcrB	B/T	15.3	17.3	18.5
4DX7_C	*E. coli*	AcrB	E/O	8.6	9.2	12.3
3AOA_A	*E. coli*	AcrB	B/T	15.3	15.9	17.9
3AOA_B	*E. coli*	AcrB	E/O	8.8	9.6	12.8
3AOA_C	*E. coli*	AcrB	A/L	12.5	15.5	17.8
3AOB_A	*E. coli*	AcrB	B/T	15	16	17.3
3AOB_B	*E. coli*	AcrB	E/O	8.6	9.4	12.5
3AOB_C	*E. coli*	AcrB	A/L	13.3	14	18
3AOC_A	*E. coli*	AcrB	B/T	15.3	16.2	18.2
3AOC_B	*E. coli*	AcrB	E/O	8.8	9.5	12.7
3AOC_C	*E. coli*	AcrB	A/L	12	14.1	17.4
3AOD_A	*E. coli*	AcrB	B/T	15.3	16.2	18
3AOD_B	*E. coli*	AcrB	E/O	9.2	9.7	12.9
3AOD_C	*E. coli*	AcrB	A/L	13.1	14.2	17.9
3NOC_A	*E. coli*	AcrB	A/L	11	15.7	16.6
3NOC_B	*E. coli*	AcrB	B/T	14.6	15.5	16.4
3NOC_C	*E. coli*	AcrB	E/O	8.5	8.9	12
3NOG_A	*E. coli*	AcrB	A/L	12.2	14.1	17.1
3NOG_B	*E. coli*	AcrB	B/T	15	16.3	17.7
3NOG_C	*E. coli*	AcrB	E/O	8.7	9.4	12.5
3W9H_A	*E. coli*	AcrB	A/L	12.2	14.6	17.6
3W9H_B	*E. coli*	AcrB	B/T	14.9	15.7	17.9
3W9H_C	*E. coli*	AcrB	E/O	9.3	9.8	12.8
4U8V_A	*E. coli*	AcrB	A/L	12.3	14.6	17
4U8V_B	*E. coli*	AcrB	B/T	14.8	16.3	17.1
4U8V_C	*E. coli*	AcrB	E/O	8.3	9.3	12.5
4U8Y_A	*E. coli*	AcrB	A/L	12.5	14.9	17.3
4U8Y_B	*E. coli*	AcrB	B/T	15.1	16.9	18.4
4U8Y_C	*E. coli*	AcrB	E/O	8.5	9.1	12.3
4U95_A	*E. coli*	AcrB	A/L	12.5	14.9	17.4
4U95_B	*E. coli*	AcrB	B/T	15.1	17	18.4
4U95_C	*E. coli*	AcrB	E/O	8.3	9.2	12.4
4U96_A	*E. coli*	AcrB	A/L	12.4	14.5	16.7
4U96_B	*E. coli*	AcrB	B/T	15.1	16.7	17.1

4U96_C	*E. coli*	AcrB	E/O	8.43	9.3	12.5
5JMN_A	*E. coli*	AcrB	A/L	11.9	14.6	17.2
5JMN_B	*E. coli*	AcrB	B/T	14.9	16.8	18.1
5JMN_C	*E. coli*	AcrB	E/O	8.3	9.1	12.2
5YIL_A	*E. coli*	AcrB	A/L	12.8	14.5	16.9
5YIL_B	*E. coli*	AcrB	B/T	14.4	14.8	17.3
5YIL_C	*E. coli*	AcrB	E/O	8.63	8.6	11
6BAJ_A	*E. coli*	AcrB	A/L	9.53	10	12.6
6BAJ_B	*E. coli*	AcrB	B/T	14.3	15.7	17.2
6BAJ_C	*E. coli*	AcrB	E/O	8.8	10.3	12.4
6SGS_A	*E. coli*	AcrB	A/L	13.5	14	16.8
6SGS_B	*E. coli*	AcrB	B/T	14.3	15.2	17.2
6SGS_C	*E. coli*	AcrB	E/O	8.66	8.94	12.1
1IWG_A	*E. coli*	AcrB	A/L	13.8	15.5	17.8
1IWG_B	*E. coli*	AcrB	A/L	13.8	15.5	17.8
1IWG_C	*E. coli*	AcrB	A/L	13.8	15.5	17.8
1OY6_A	*E. coli*	AcrB	A/L	13.7	15	17.7
1OY6_B	*E. coli*	AcrB	A/L	13.7	15	17.7
1OY6_C	*E. coli*	AcrB	A/L	13.7	15	17.7
1OY8_A	*E. coli*	AcrB	A/L	13.7	14.8	17.4
1OY8_B	*E. coli*	AcrB	A/L	13.7	14.8	17.4
1OY8_C	*E. coli*	AcrB	A/L	13.7	14.8	17.4
1OY9_A	*E. coli*	AcrB	A/L	13.6	14.9	17.8
1OY9_B	*E. coli*	AcrB	A/L	13.6	14.9	17.8
1OY9_C	*E. coli*	AcrB	A/L	13.6	14.9	17.8
1OYD_A	*E. coli*	AcrB	A/L	13.8	15	17.8
1OYD_B	*E. coli*	AcrB	A/L	13.8	15	17.8
1OYD_C	*E. coli*	AcrB	A/L	13.8	15	17.8
2DHH_A	*E. coli*	AcrB	B/T	15.2	15.7	17.7
2DHH_B	*E. coli*	AcrB	E/O	9	9.4	13
2DHH_C	*E. coli*	AcrB	A/L	12.9	15.8	18.3
2DR6_A	*E. coli*	AcrB	B/T	15.1	15.6	17.7
2DR6_B	*E. coli*	AcrB	E/O	9.2	10.1	13.3
2DR6_C	*E. coli*	AcrB	A/L	13.1	15.9	18.2
2DRD_A	*E. coli*	AcrB	B/T	15.2	15.8	17.8
2DRD_B	*E. coli*	AcrB	E/O	9	9.5	13
2DRD_C	*E. coli*	AcrB	A/L	12.9	15.9	18.3
2GIF_A	*E. coli*	AcrB	A/L	13.9	14.2	17.7
2GIF_B	*E. coli*	AcrB	B/T	15.2	15.4	18.4
2GIF_C	*E. coli*	AcrB	E/O	9.3	8.5	11.6
2HRT_A	*E. coli*	AcrB	A/L	14.2	14.8	17.9
2HRT_B	*E. coli*	AcrB	B/T	14.6	15.1	17.9
2HRT_C	*E. coli*	AcrB	E/O	9.1	9	11.9
2J8S_A	*E. coli*	AcrB	A/L	12.7	14.8	17.7
2J8S_B	*E. coli*	AcrB	B/T	15.1	16	17.8
2J8S_C	*E. coli*	AcrB	E/O	8	9.1	12.6

2V50_A	*P. aeruginosa*	MexB	A/L	11	9.18	11.6
2V50_B	*P. aeruginosa*	MexB	B/T	13.67	12.93	15.14
2V50_C	*P. aeruginosa*	MexB	E/O	8.04	8.17	10.6
3W9I_A	*P. aeruginosa*	MexB	A/L	11.7	10.6	13.2
3W9I_B	*P. aeruginosa*	MexB	B/T	13	13	14.8
3W9I_C	*P. aeruginosa*	MexB	E/O	8.6	8.6	10.9
3W9J_A	*P. aeruginosa*	MexB	A/L	10.9	10.1	12.3
3W9J_B	*P. aeruginosa*	MexB	B/T	13.4	12.3	15.2
3W9J_C	*P. aeruginosa*	MexB	E/O	8.8	9.1	11.3
6T7S_A	*P. aeruginosa*	MexB	B/T	12.6	12	14.3
6T7S_B	*P. aeruginosa*	MexB	E/O	9	8.8	12
6T7S_C	*P. aeruginosa*	MexB	A/L	11.5	10.9	13.3
5T0O_A	*C. jejuni*	CmeB	E/O	9.6	9.2	12.7
5T0O_B	*C. jejuni*	CmeB	E/O	10.5	9.4	12.4
5T0O_C	*C. jejuni*	CmeB	E/O	9.4	9.3	12.8
5LQ3_A	*C. jejuni*	CmeB	E/O [R]	10.9	10.1	13
5LQ3_B	*C. jejuni*	CmeB	B/T	15.4	14	15.7
5LQ3_C	*C. jejuni*	CmeB	E/O	9.9	9.4	12.3
6VKS_A	*N. gonorrhoeae*	MtrD	A/L	14.1	14.3	16.8
6VKS_B	*N. gonorrhoeae*	MtrD	B/T	15.7	15.2	17.2
6VKS_C	*N. gonorrhoeae*	MtrD	E/O	9.8	9.7	13
6VKT_A	*N. gonorrhoeae*	MtrD	A/L	16	15.2	17.4
6VKT_B	*N. gonorrhoeae*	MtrD	B/T	17.2	16.6	18.6
6VKT_C	*N. gonorrhoeae*	MtrD	E/O	10.1	9.2	12.5
6OWS_A	*A. baumannii*	AdeB	E/O	9	9.1	12
6OWS_B	*A. baumannii*	AdeB	E/O	9	9.1	12
6OWS_C	*A. baumannii*	AdeB	E/O	9	9.1	12
7CZ9_A	*K. pneumoniae*	OqxB	B/T	12	11.2	12.1
7CZ9_B	*K. pneumoniae*	OqxB	B/T	11.6	11	12.2
7CZ9_C	*K. pneumoniae*	OqxB	B/T	12.2	11.4	12.4
7CZ9_D	*K. pneumoniae*	OqxB	B/T	12.5	11.8	12.8
7CZ9_E	*K. pneumoniae*	OqxB	B/T	11.6	11	12.1
7CZ9_F	*K. pneumoniae*	OqxB	B/T	11.4	10.4	11.2

*****Access/Loose (A/L), Binding/Tight (B/T), and Extrusion/Open (E/O). Distances measured by cα atoms between the residues and shown in Å. In *E. coli* AcrB the D1 is between R717 and M662, D2 is between R717 and F664, D3 is R717 and F666. In *P. aeruginosa* MexB the D1 is between R716 and M662, D2 is between R716 and F664, D3 is R717 and F666. In *C. jejuni* CmeB the D1 is between R711 and S657, D2 is between R711 and V659, D3 is R711 and I661. In *N. gonorrhoeae* MtrD the D1 is between R714 and F658, D2 is between R714 and I660, D3 is R714 and V662. In *A. baumannii* AdeB the D1 is between W708 and E654, D2 is between W708 and M656, D3 is W708 and V658. In *K. pneumoniae* OqxB the D1 is between I721 and F667, D2 is between I721 and F669, D3 is I721 and I671.

Supplementary Table 9: Bacterial Strains

Strains	Reference or Source
E. coli K-12 BW25113 (Wild Type)	3
E. coli K-12 BW25113 *ΔacrB::kan*	3
E. coli C43(DE3) (Wild Type)	4
E. coli C43(DE3) *ΔacrB::kan*	This Study
E. coli K-12 BW25113 *ΔacrA ΔacrB::kan*	This Study
E. coli C43(DE3)ΔacrB carrying pET21a	This Study
E. coli C43(DE3)ΔacrB carrying pEcoB = (+ *acrB*)	This Study
E. coli C43(DE3)ΔacrB carrying pOqxB = (+ *oqxB*)	This Study
E. coli K-12 BW25113 carrying pTrc99a	This Study
E. coli K-12 BW25113 carrying pEcoAB = (+ *acrA* + *acrB*)	This Study
E. coli K-12 BW25113 carrying pOqxA*B* = (+ *oqxA* + *oqxB*)	This Study
E. coli K-12 BW25113 carrying pOqxA-OqxBR157A	This Study

Supplementary Table 10: Plasmids and Constructs used

Plasmids	Description	Reference or Source
pET21a	Cloning Vector	Invitrogen
pTrc99a	Cloning Vector	Genscript
pEcoB	pET21a carrying His_6-Tagged *E. coli acrB*; Amp^r	This Study
pOqxB	pET21a carrying His_6-Tagged *K. pneumoniae oqxB*; Amp^r	This Study
pEcoAB	pTrc99a derivative carrying *E. coli acrA* & *acrB*; Amp^r	This Study
pOqxAB	pTrc99a derivative carrying *K. pneumoniae oqxA* & *oqxB*; Amp^r	This Study
pOqxA-OqxBR157A	pTrc99a derivative carrying *K. pneumoniae oqxA* & *oqxB* with mutation R157A; Amp^r	This Study

Supplementary Table 11: Cloning Primers for PCR amplification of Inserts (Single Expression Complementation)

Insert Amplified	Primers	Primer Sequence (5' → 3')	Restriction Endonuclease (RE)
E. coli acrB	Eco_acrB_FP	5' ACGCCATATGATGCCTAATTTCTTTATC 3'	NdeI
	Eco_acrB_RP	5' AGCGCTCGAGATGATGATCGACAGTATGGC 3'	XhoI
K. pneumoniae oqxB	Kpn_oqxB_FP	5' ACGCCATATGGACTTTTCCCGCTTTTTTA 3'	NdeI
	Kpn_oqxB_RP	5' AGCGCTCGAGGGCGGGCAGATCCTCCTGGA 3'	XhoI

Supplementary Table 12: Cloning Primers for PCR amplification of Inserts (Double Expression complementation)

Insert Amplified	Primers	Primer Sequence (5' → 3')	Restriction Endonuclease (RE)
E. coli acrA-acrB	Eco_acrAB_FP	5'GATACATATGCTGTTTACGATTAATCATCCGGCTCGTATAATGTGTGGTCACACAGGAAACAGACCATGAACAAAAACAGAGGGTTTAC 3'	**NdeI**
	Eco_acrAB_RP	5' CCCAAGCTTTCAATGATGATCGACAGTATG 3'	**HindIII**
K. pneumoniae oqxA-oqxB	Kpn_oqxAB_FP	5'GATACATATGGAGCTGTTTACGATTAATCATCCGGCTCGTATAATGTGTGGTCACACAGGAAACAGACCATGAGCCTGCAAAAAACCTGGGGAAACAT 3'	**NdeI**
	Kpn_oqxAB_RP	5' CCCAAGCTTCTAGGCGGGCAGATCCTCCTGGAC 3'	**HindIII**

Supplementary Table 13: Primers for OqxB: R157A Site Directed Mutagenesis

Primers	Sequence
OqxB R157A FP	5’ GCTGTATATGGCGAACTACGCCACGCTG 3’
OqxB R157A RP	5’ CGTGGCGTAGTTCGCCATATACAGCGAGTC 3’

Supplementary Table 14: Primer pairs for quantitative RT-PCR.

Species	Gene Name	Product Size (in bp)	Primer	Primer Sequence
E. coli	*acrB*	214	acrB_RT_FP_2	5’ CGTTGATTCCGACCATTGCC 3’
			acrB_RT_RP_2	5’ CCCATCGACTTACGGGTAGC 3’
	dnaK	130	dnaK_RT_Fwd	5’GTGCGAAACTGGAAAGCCT 3’
			dnaK_RT_Rev	5’GAGTCTGACCACCAACGAGG 3’
K. pneumoniae	*oqxB*	267	oqxB_RT-2_FP	5’ATCGACAGGCCGATTTTCGC 3’
			oqxB_ RT-2_RP	5’GAAGGTGACGGTGGTGACCAGC 3’

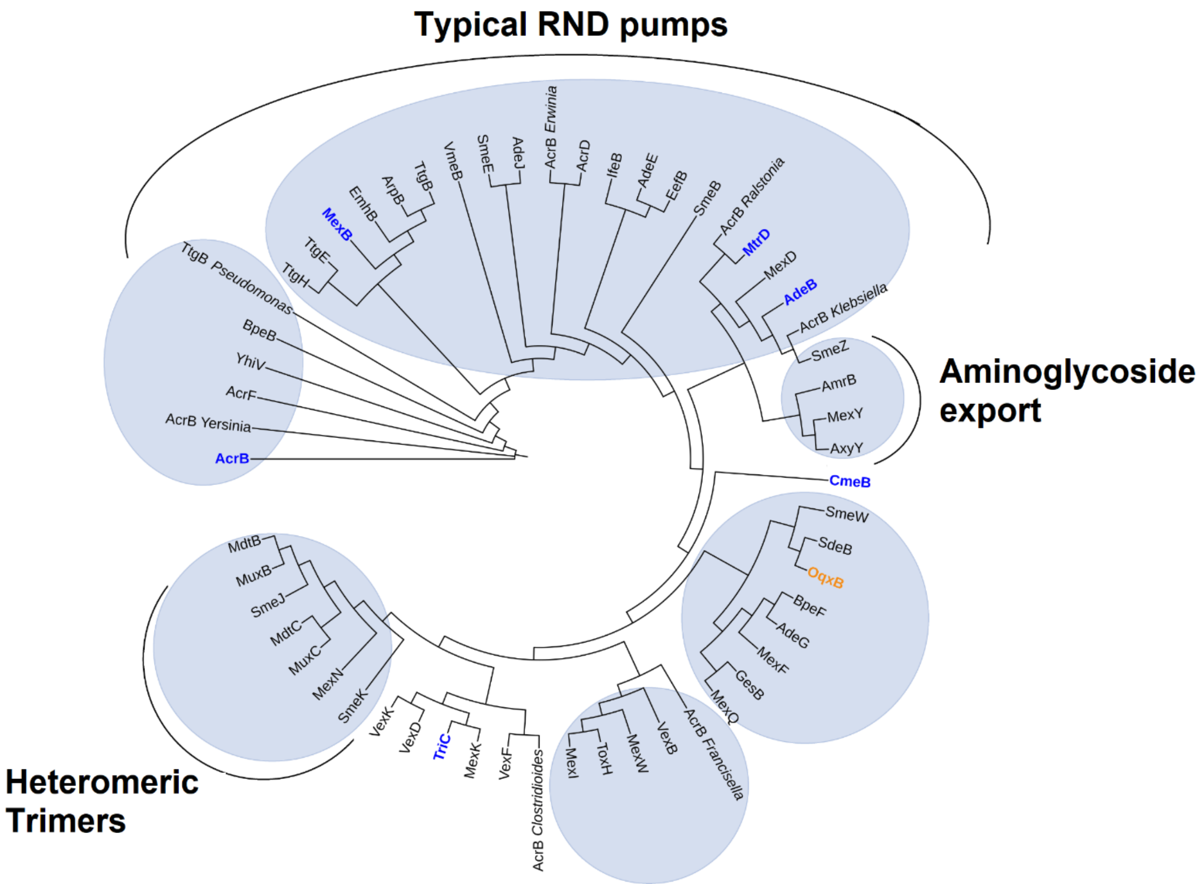

Supplementary Figure 1: The figure depicting the diversity of various RND efflux pumps. The multi-drug resistance influencing OqxB (labelled in orange) is distant from typical RND efflux pump. Structurally characterised members are labelled in blue. The list of RND transporters considered from the 2.A.6.2.xx [categorised as the Hydrophobe/Amphiphile Efflux-1 (HAE1) Family] transporter classification database by Milton Saier, UCSD (http://www.tcdb.org/search/result.php?tc=2.A.6) to generate phylogenetic tree.

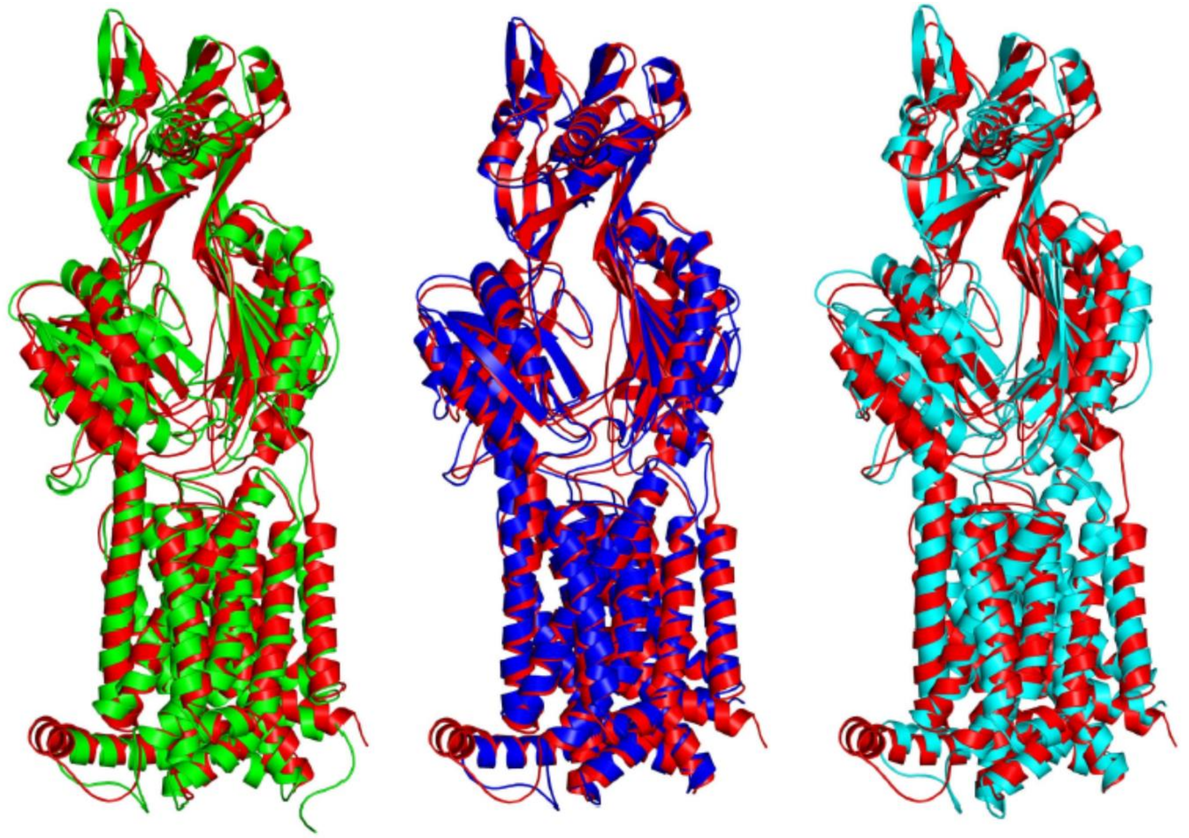

Supplementary Figure 2: Structural comparison of OqxB protomer with AcrB. Superposition of OqxB protomer (red colour cartoon) on Access protomer (green colour cartoon), Binding protomer (blue colour cartoon), and Extrusion protomer (cyan colour cartoon) of AcrB asymmetric trimer (PDB ID:4DX5).

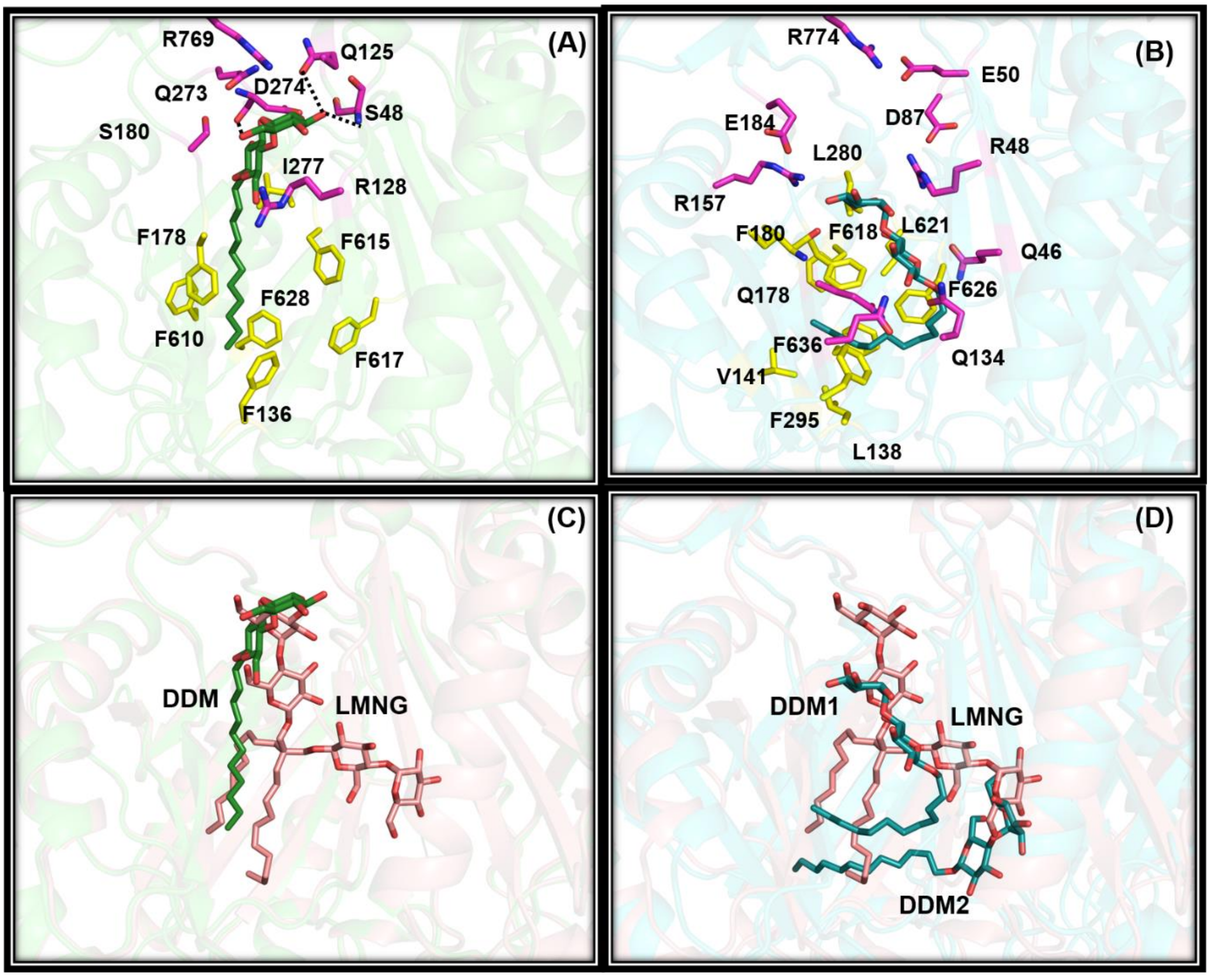

Supplementary Figure 3: Binding mode differences of DDM and LMNG molecules. Experimentally determined DDM binding mode with MexB (A) and OqxB (B) structures compared. MexB binding protomer (PDB ID:3W9I) superimposed on OqxB protomer to capture the differences. The LMNG bound MexB binding protomer (PDB ID: 6IIA) superposed on DDM bound MexB structure (C) and OqxB (D) to show the variations. Hydrophobic residues are shown as yellow sticks whereas hydrophilic residues depicted as magenta sticks and labelled accordingly. MexB bound DDM molecule shown as green, OqxB bound DDM molecules shown as cyan, and LMNG molecule as pale magenta colour sticks.

In MexB crystal structure, the two sugar rings of DDM molecule protrude into hydrophilic pocket and forms hydrogen bond interactions with Q125 side chain, S48 main chain NH and D274 main chain carbonyl (Supplementary **Figure 3A**). The OqxB DDM1 molecule does not have such deep penetration compared to MexB DDM molecule (Supplementary **Figure 3B**). The LMNG compound binds to MexB such that the two sugar moieties of LMNG overlaps on sugar rings of DDM molecule, while the remaining two rings bind perpendicularly (Supplementary **Figure 3C**) overlapping with the two sugar rings of DDM2 molecule of OqxB. The DDM2 molecule occupies a secondary pocket (other than conventional DDM sugar binding pocket located near exit funnel) similar to LMNG molecule (Supplementary **Figure 3D**).

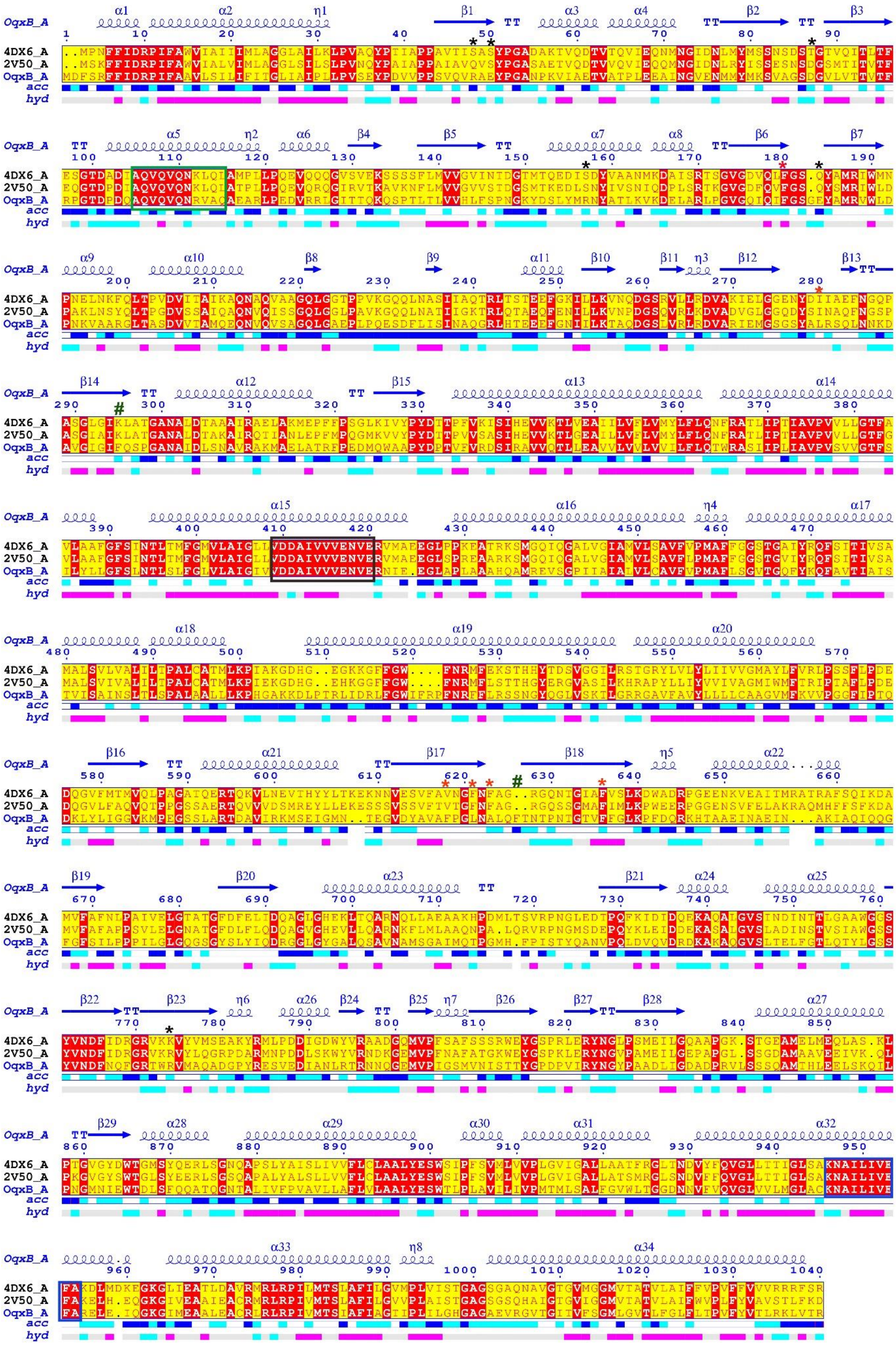
OqxB_A
4DX6_A
2V50_A
OqxB_A
acc
hyd

Supplementary Figure 4: Structure-based sequence alignment of OqxB with other RND pumps. The OqxB sequence aligned with AcrB (4DX6_A) and MexB (2V50_A) using ENDscript webserver (https://espript.ibcp.fr/ESPript/cgi-bin/ENDscript.cgi) to compare the similarities and differences. Important hydrophobic residues and hydrophilic residues of OqxB substrate-binding pocket highlighted with red and black asterisks, respectively. Aromatic residues which are unique in OqxB substrate-binding pocket are emphasised with green colour #. The conserved central α-helix, TM4 and TM10 are highlighted with green, black, and blue boxes, respectively.

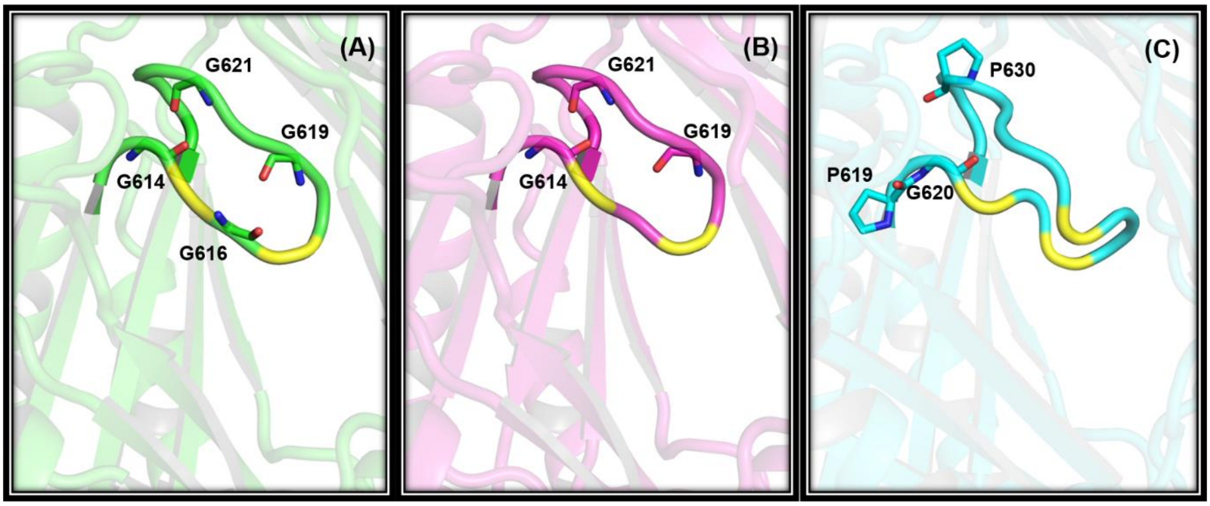

Supplementary Figure 5: Residual composition of g-loop. The glycine or proline residues present on AcrB (A), MexB (B) and OqxB (C) of g-loop are highlighted. These residues shown as sticks and residue numbers labelled. Important hydrophobic residues which are crucial for substrate binding also highlighted with yellow colour.

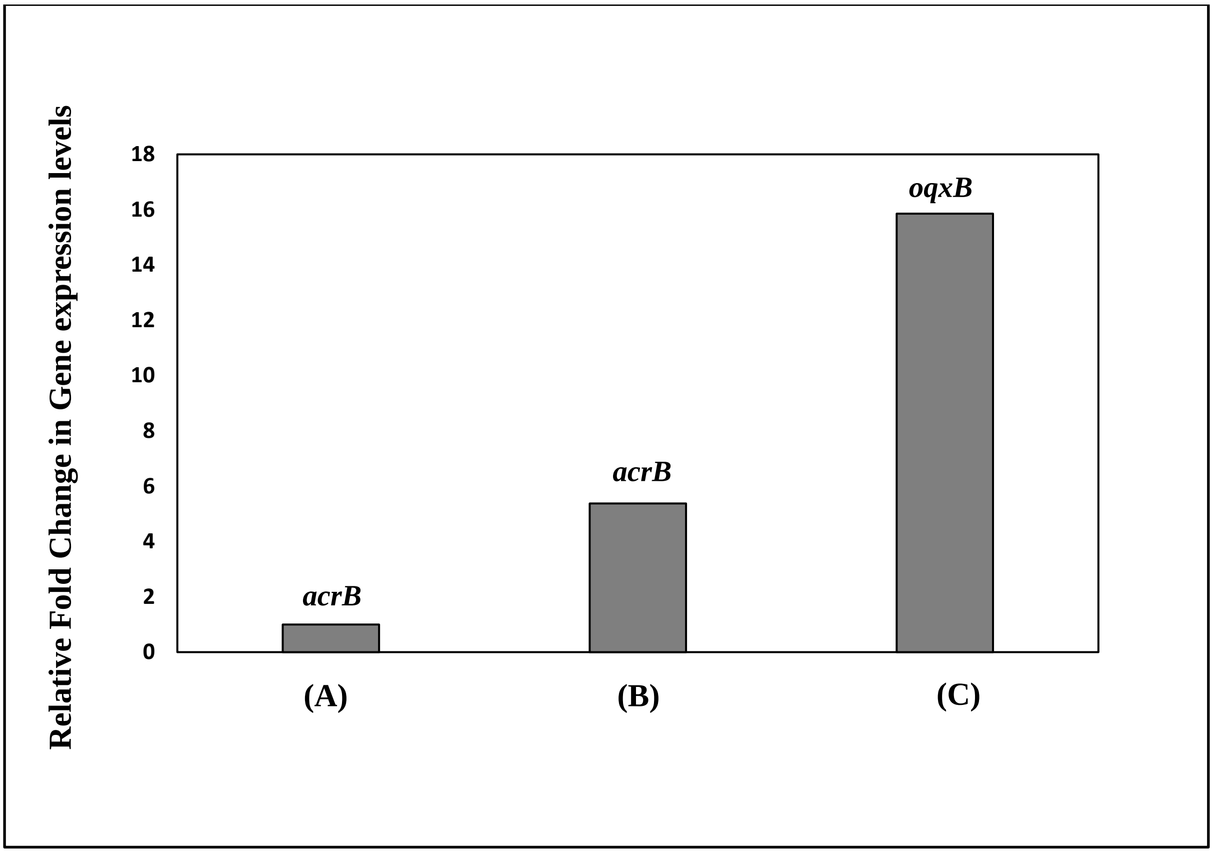

Supplementary Figure 6: mRNA estimation by quantitative RT-PCR. The relative expression levels for the genes *acrB* and *oqxB* in each of the overexpression systems, (A) *E. coli K-12* BW25113 wildtype, BW(WT) ; (B*)* WT carrying pEcoA*B* (overexpressing *acrA*-*acrB*) and (C) WT carrying pOqxAB (overexpressing *oqxA*-*oqxB*), was estimated by quantitative Real Time PCR (qRT-PCR). Each point represents the ratios of the fold change for each of the target genes (*acrB* and *oqxB*), relative to the expression level of *acrB* in *E. coli* wild type system, without any complementation, as observed from two independent biological experiments (n=2) in technical triplicates. (Refer to Supplementary **Table 9** and **Table 10,** for details of the strains and constructs respectively).

Supplementary Table 15: Ct Values for the quantification of *acrB* and *oqxB* overexpression by qRT-PCR

Strain	Gene	Ct1	Ct2	Ct3	Average Ct	ΔCt (Ct_{gene}-Ct_{dnaK})	Fold Change = 2^-ΔCt Normalization w.r.t. DnaK	Relative Expression levels w.r.t. WT AcrB
BW25113 (WT)	*acrB*	22.06	21.86	21.92	21.95	5.20	0.03	1
	dnaK	16.80	16.78	16.68	16.75	0.00	1.00	
BW25113 +pAcrAB	*acrB*	20.44	20.31	20.29	20.35	2.77	0.15	5.38
	dnaK	17.68	17.60	17.46	17.58	0.00	1.00	
BW25113 +pOqxAB	*oqxB*	13.71	13.38	13.08	13.39	1.21	0.43	15.85
	dnaK	12.57	12.20	11.77	12.18	0.00	1.00	

Ct1, Ct2, Ct3: Ct values for technical triplicates

Over-expression of AcrB and OqxB in *E. coli* Wild Type complementation systems:

The overexpression of the different efflux pump components AcrB and OqxB were confirmed by estimating the mRNA expression of these genes in the three strains: *E. coli K-12* BW25113 wildtype (WT), and its overexpressing counterparts for AcrAB and OqxAB. The Ct values were compared and normalized with the house-keeping gene *dnaK*, which is constitutively overexpressed around 38000 copies per cell[1]. The expression levels for each of the genes were then estimated using the relative Ct method, as discussed elsewhere[2]. The relative fold change in the expression levels for these genes were than compared with respect to the *acrB* levels in BW25113 (WT). Upon comparison, the quantification of the target gene mRNA revealed a 5-6-fold increase in the expression of AcrB in the WT with pAcrAB strain, and a 2.5-fold increase of OqxB levels in the WT with pOqxAB strain, as compared to the levels of AcrB in the WT with pAcrAB strain (Supplementary **Table 15** and Supplementary **Figure 6**). Overall, these values confirm the overexpression of AcrB and OqxB in the recombinant expression system in *E. coli.*

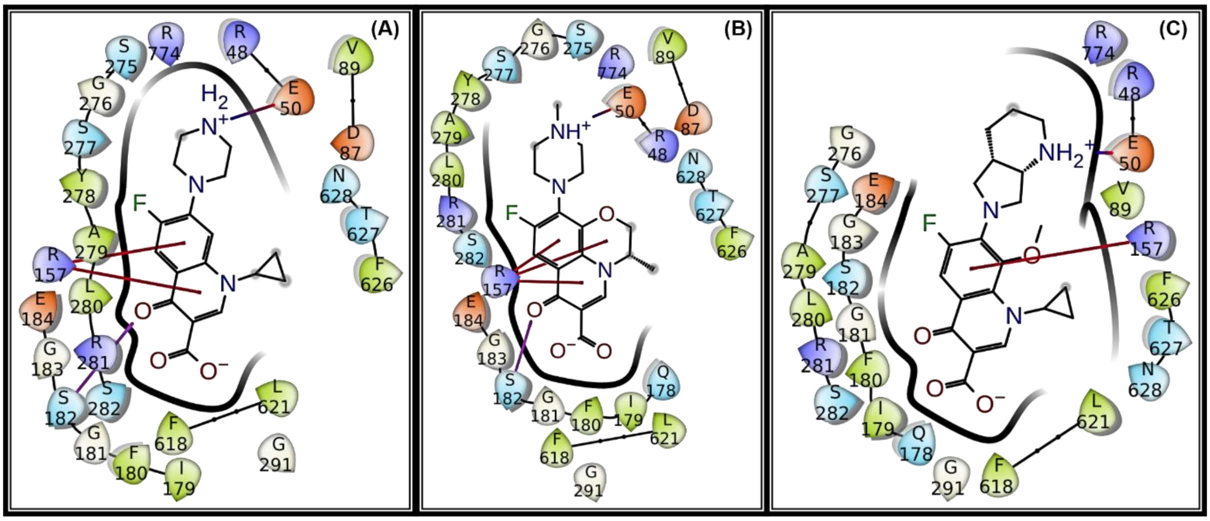

Supplementary Figure 7: Fluroquinolone's interaction diagrams with OqxB. 2D protein-ligand interaction fingerprints of ciprofloxacin (A), levofloxacin (B), and moxifloxacin (C) generated using molecular docking predicted binding modes. Inter-molecular hydrogen bond interactions shown as magenta arrows (pointed arrow towards acceptor atom) and pi-cation interaction between R157 side chain and quinolone ring depicted as solid red line.

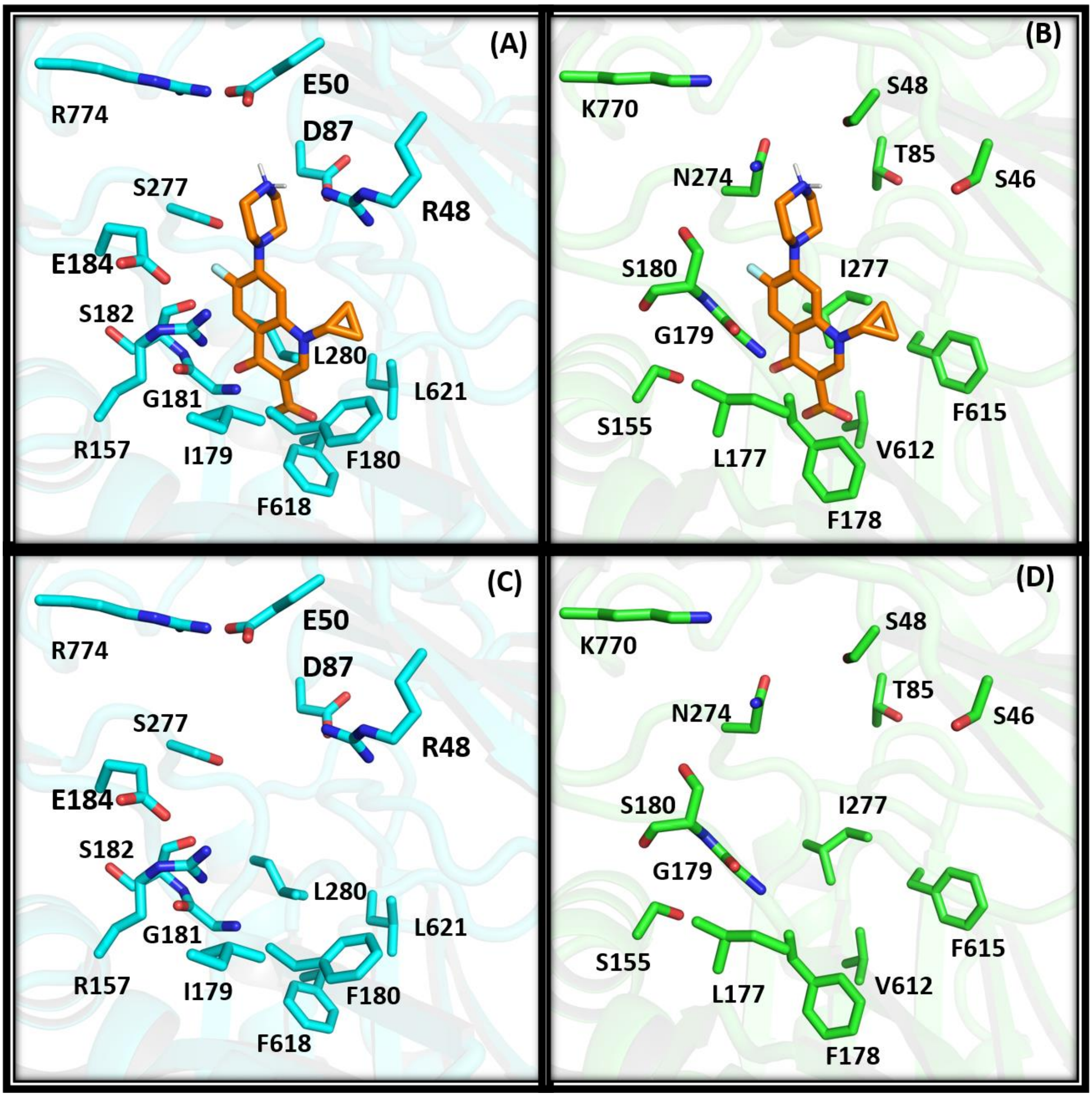

Supplementary Figure 8: Ciprofloxacin binding pocket residues comparison. Comparison of ciprofloxacin binding pocket residues of OqxB (A) and AcrB (B). AcrB binding protomer (PDB ID: 4DX5) considered for superposition on OqxB:ciprofloxacin complex (binding mode determined by docking simulations). Ciprofloxacin shown as orange sticks and binding pocket residues of OqxB shown as cyan sticks and AcrB residues shown as green sticks. The bound ciprofloxacin removed from OqxB (C) and AcrB (D) for close view of binding pocket residues.

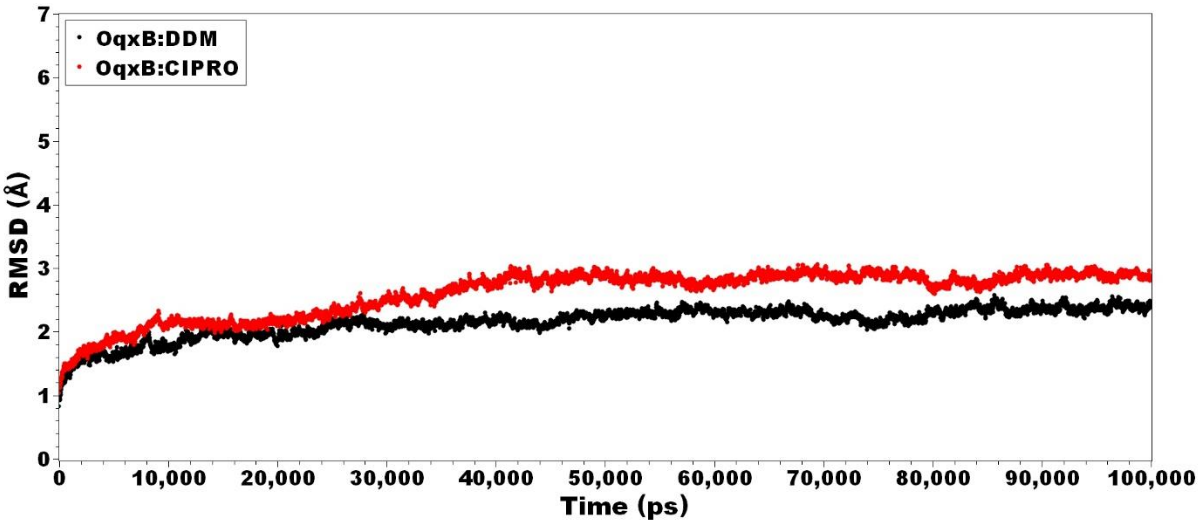

Supplementary Figure 9: RMSD analyses of 100ns MD simulations. MD simulations analyses of DDM as well as ciprofloxacin (CIPRO) with OqxB trimer. The root-mean square deviation for total 100ns simulation shown in Å on y-axis whereas x-axis represent time in picoseconds.

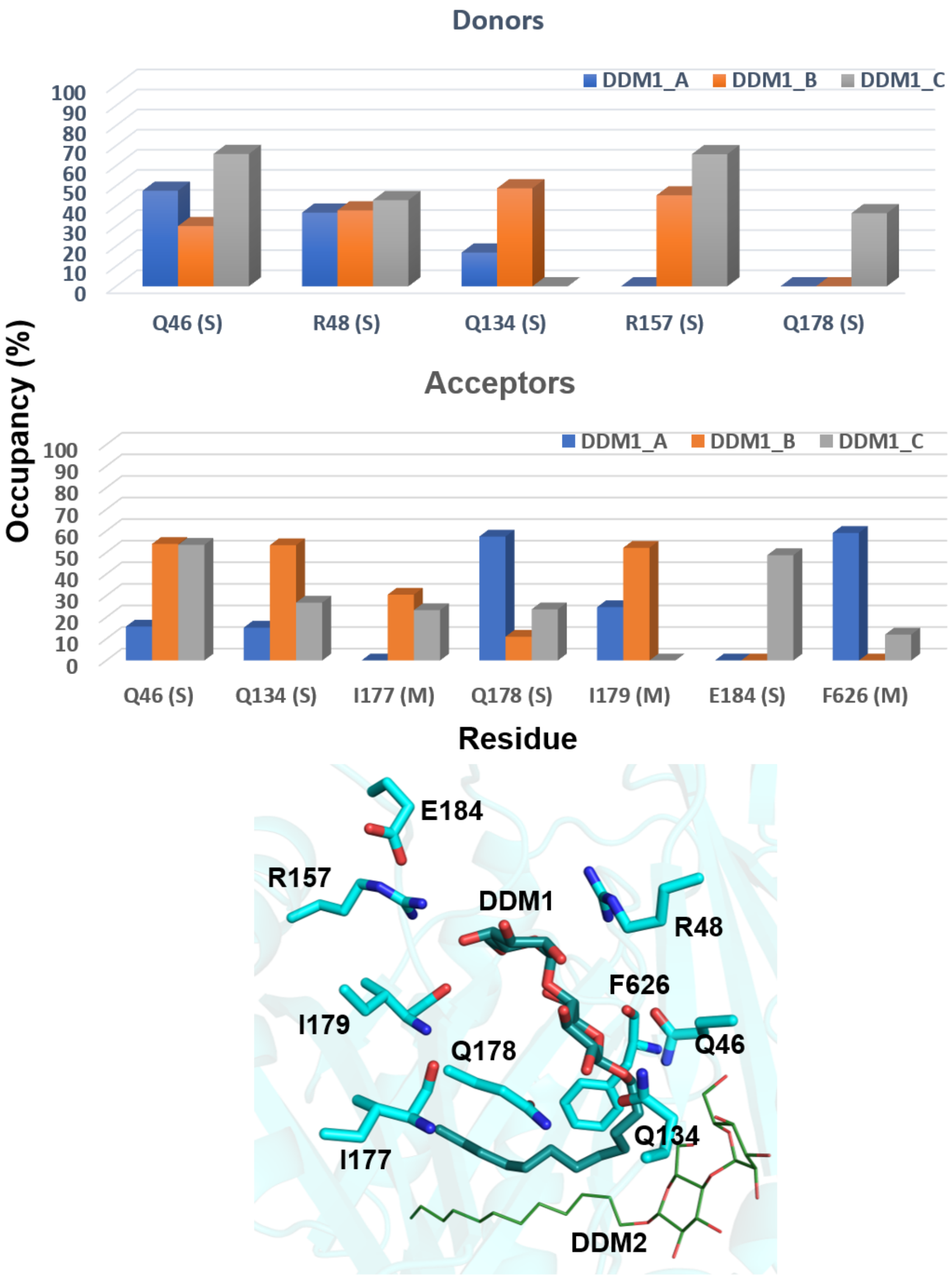

Supplementary Figure 10: Interaction pattern of DDM1. The hydrogen bond occupancy (%) rate of DDM molecules with hydrogen bond donor atoms as well as acceptor atoms of substrate-binding pocket were analysed. Individual DDM1 molecules considered for analysis and compared. Interacting atom from side chain represented as (S) whereas from main chain atom (M) for clarity. All the interacting residues depicted as sticks including DDM1 molecule. The DDM2 molecule shown as green colour lines.

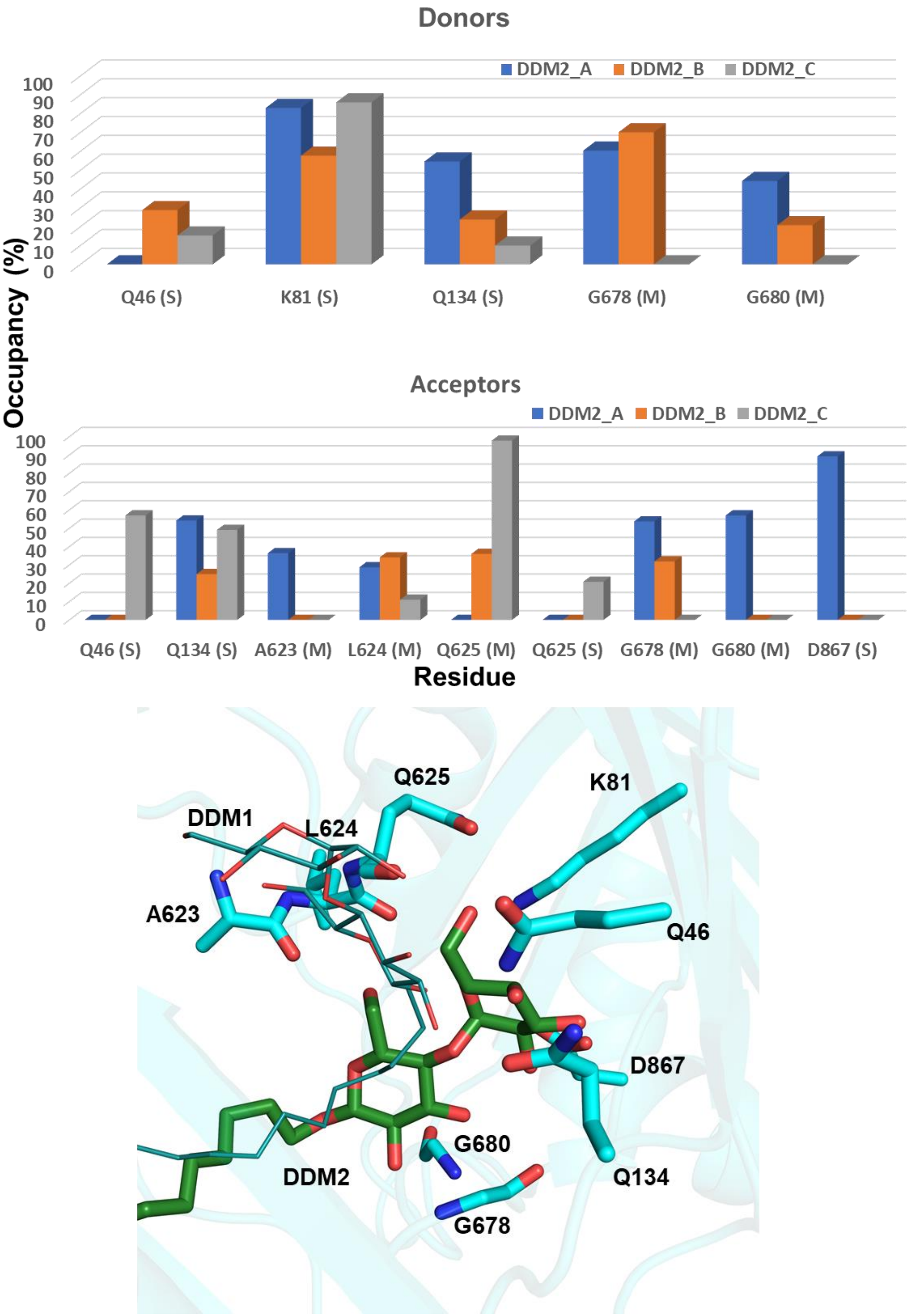

Supplementary Figure 11: Interaction pattern of DDM2. The hydrogen bond occupancy (%) rate of DDM molecules with hydrogen bond donor atoms as well as acceptor atoms of substrate-binding pocket were analysed. Individual DDM2 molecules considered for analysis and compared. Interacting atom from side chain represented as (S) whereas from main chain atom (M) for clarity. All the interacting residues depicted as sticks including DDM2 molecule. The DDM1 molecule shown as cyan colour lines.

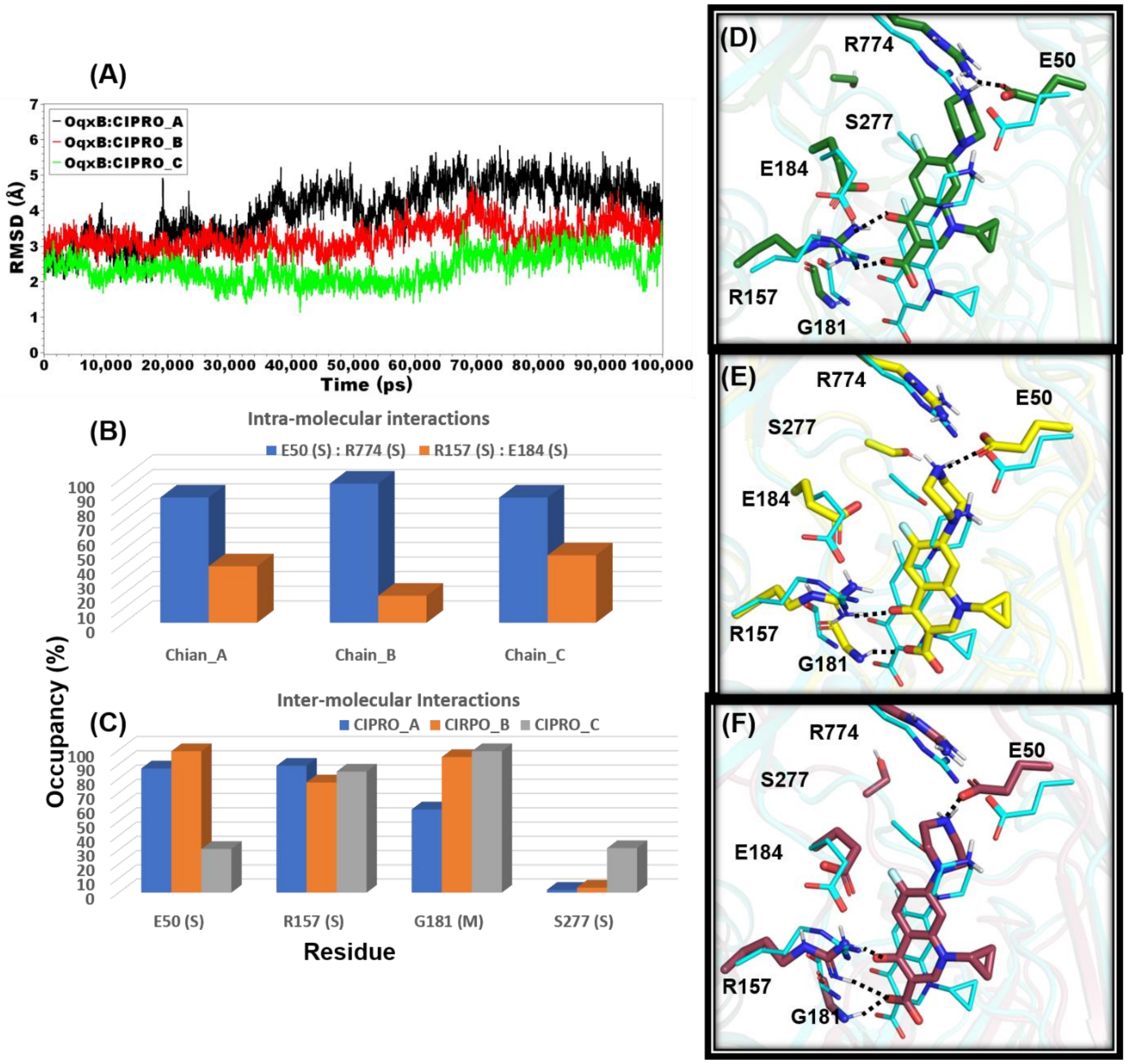

Supplementary Figure 12: Interaction pattern of ciprofloxacin with OqxB. The ligand RMSDs (A) of three ciprofloxacin molecules measured in comparison with initial minimized orientation of three ciprofloxacin molecules. Two key intra-molecular hydrogen bond interactions measured in all the three protomers and % occupancies presented (B). Inter-molecular hydrogen bond interaction occupancies presented as bar graph for three individual CIPRO molecules (C). Initial (minimized structure) and final snapshot of MD simulations of CIPRO molecules of chain_A (D), chain_B (E) and chain_C (F). Initial snapshot depicted with cyan cartoon and CIPRO as well as important interacting residues shown as cyan colour lines. Final snapshot CIPRO molecules and key residues shown as sticks. Inter-molecule hydrogen bond interactions depicted as broken lines.

The main chain NH of G181 residue also exhibited hydrogen bond interaction with CIPRO carboxyl oxygen atom in two protomers while one protomer (chain A), had comparatively less hydrogen bond occupancy. Moreover, CIPRO molecule showed only 30% interaction occupancy with E50 residue side chain in one protomer (chain C) unlike other two protomers. To understand these differences in interactions pattern between the protomers, we compared (Supplementary **Figure 12D, 12E, and 12F**) the final snapshot of three individual protomers with their respective initial snapshot (docking predicted binding mode of CIPRO). The three CIPRO molecules moved towards hydrophilic pocket near to the exit funnel which is marginally high in first protomer (chain A) leading to high ligand RMSD and thereby losing hydrogen bond interaction with G181 residue (Supplementary **Figure 12A and 12D**). Such ligand shift was not observed in third protomer (chain C) until 70ns as the positively charged piperazine showed hydrogen bond interaction with S277 side chain oxygen atom (Supplementary **Figure 12C**). This is also evident from ligand RMSD analyses where CIPRO showed within 2.5Å variation until 70ns simulation time.

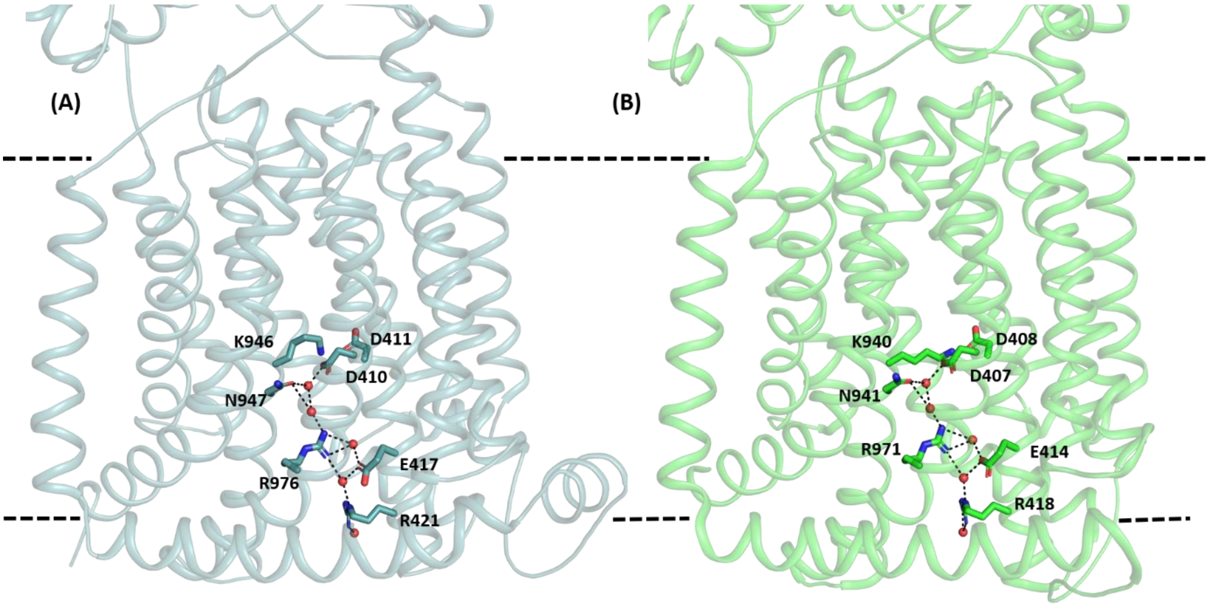

Supplementary Figure 13: OqxB & AcrB proton pathway. Comparison of OqxB (A) and AcrB (B) inner membrane docking domain with important residues which are involved in the proton transport pathway. Five water molecules depicted as red spheres and the hydrogen bond interactions highlighted with broken lines. The residues shown as sticks and labelled. All the residues and interactions are conserved in both the pumps. The B chain (binding protomer) from *E. coli* AcrB crystal structure (PDB ID: 4DX5) considered for comparison.

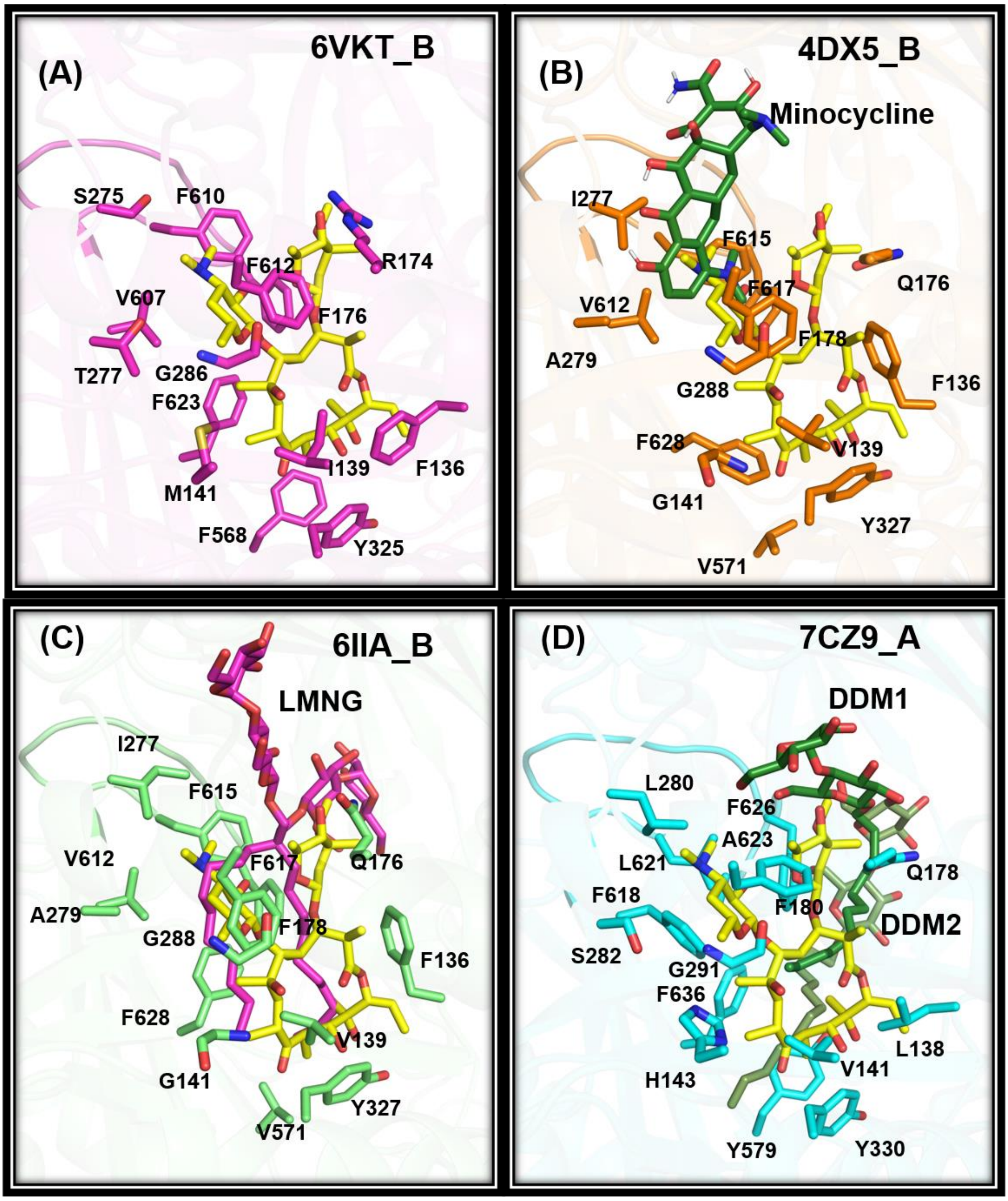

Supplementary Figure 14: Comparison of probable proximal binding pocket. Erythromycin bound *Neisseria gonorrhoeae* (PDB ID: 6VKT) binding protomer (A) considered for comparison by superposition method. Minocycline bound to *E. coli* AcrB (B), LMNG bound to MexB (C) as well as DDM molecules bound to OqxB (D) are considered for comparative studies. Erythromycin molecule shown as yellow sticks whereas minocycline, LMNG and DDM molecules depicted as dark green, magenta, and green sticks, respectively. 5Å around erythromycin molecule are considered as proximal pocket residues which are depicted as sticks and labelled accordingly.

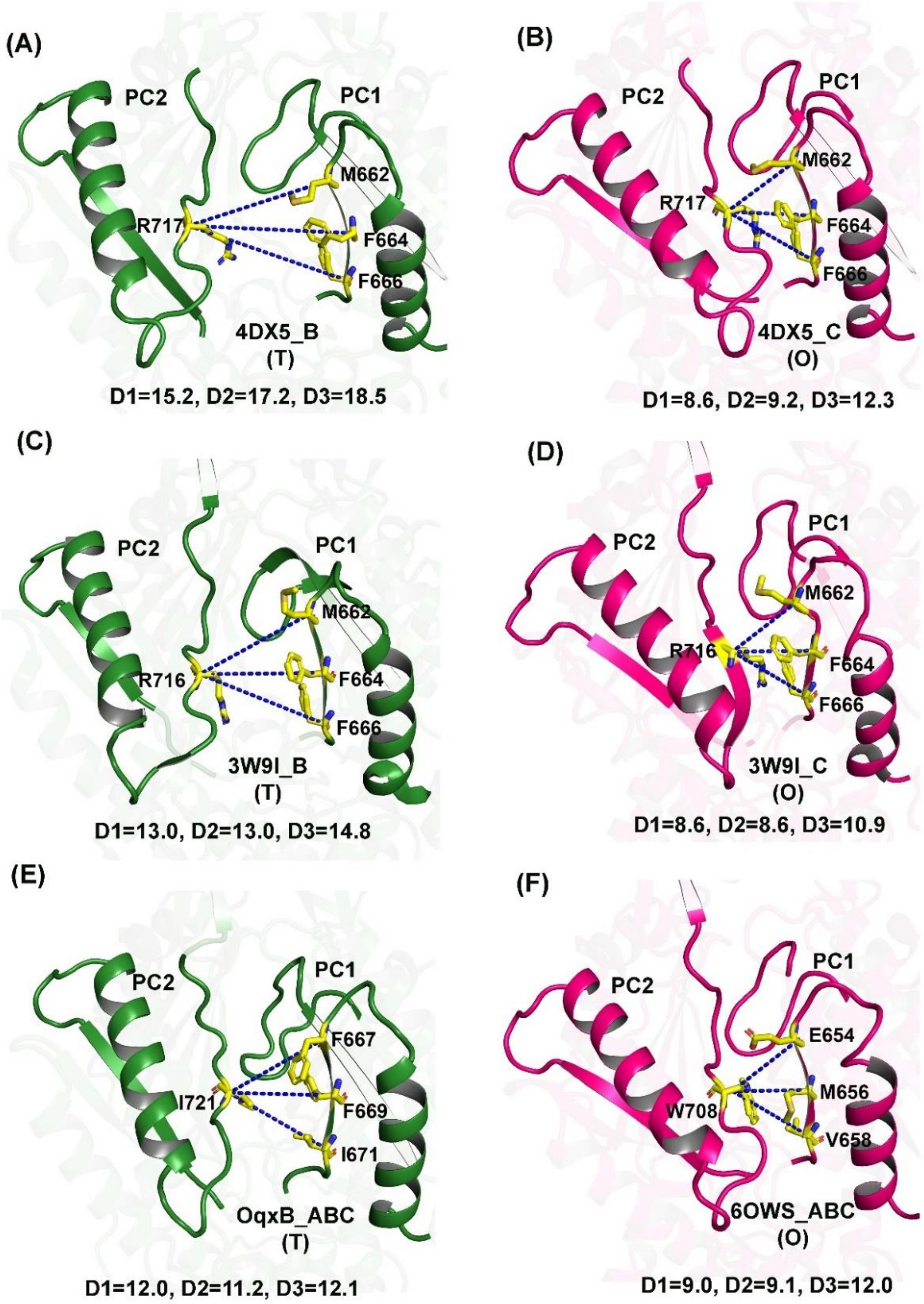

Supplementary Figure 15: Distance measurement between PC1 and PC2 sub-domain residues. Distances shown for D1, D2 and D3 are in Å. In *E. coli* AcrB (PDB ID: 4DX5) the distances D1 is between R717 and M662, D2 is between R717 and F664, D3 is R717 and F666 measured in the binding/tight protomer (A), extrusion/open protomer (B). In *P. aeruginosa* MexB (PDB ID: 3W9I) the distances D1 is between R716 and M662, D2 is between R716 and F664, D3 is R717 and F666 measured in the binding/tight protomer (C), extrusion/open protomer (D). In *K. pneumoniae* OqxB the distances D1 is between I721 and F667, D2 is between I721 and F669, D3 is I721 and I671 measured in the binding/tight protomers (E). In *A. baumannii* AdeB (PDB ID: 6OWS) the distances D1 is between W708 and E654, D2 is between W708 and M656, D3 is W708 and V658 measured in the extrusion/open protomers (F). The Cα atoms were considered for measurement.

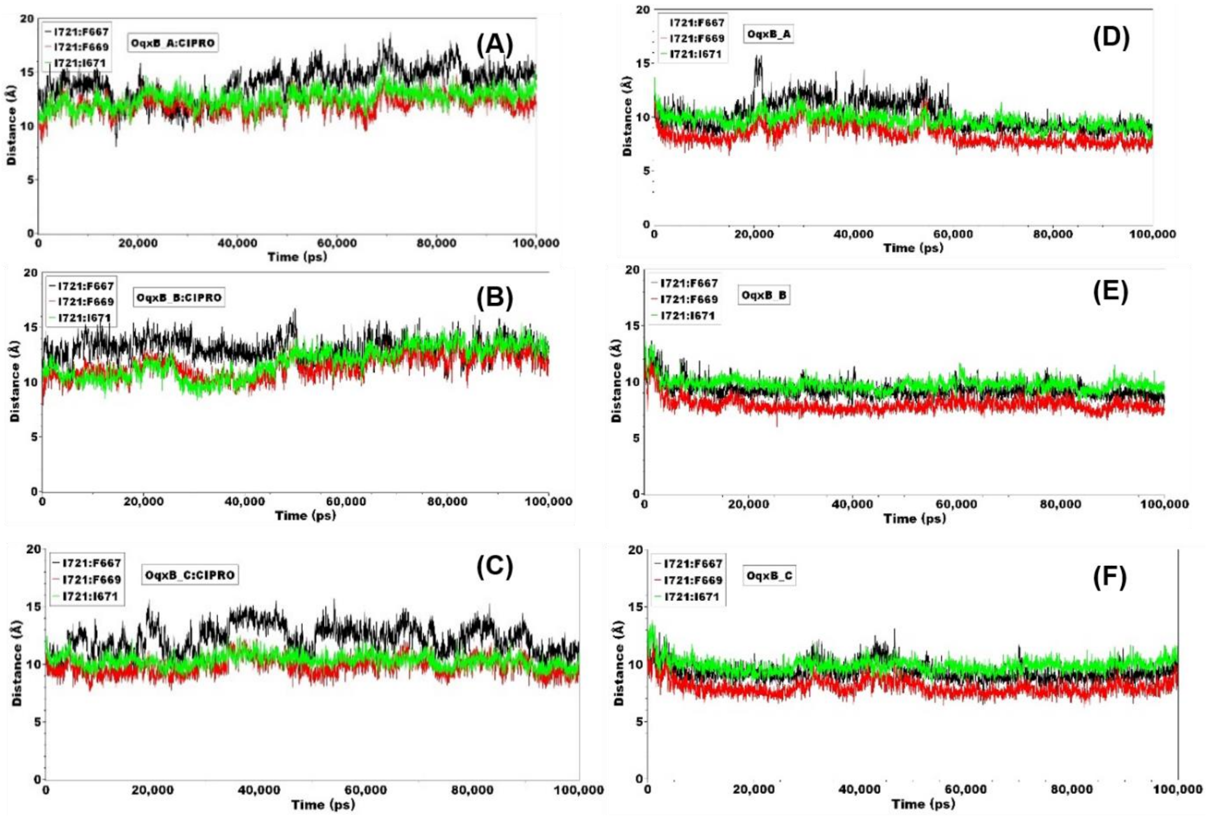

Supplementary Figure 16: Distance measurement between PC1 and PC2 sub-domain residues from MD simulation trajectories. In the presence of ligand (ciprofloxacin) the distances are in between 12 Å and 15 Å (A, B and C) whereas the distances oscillated around 10Å in case of unliganded OqxB simulations (D, E and F).

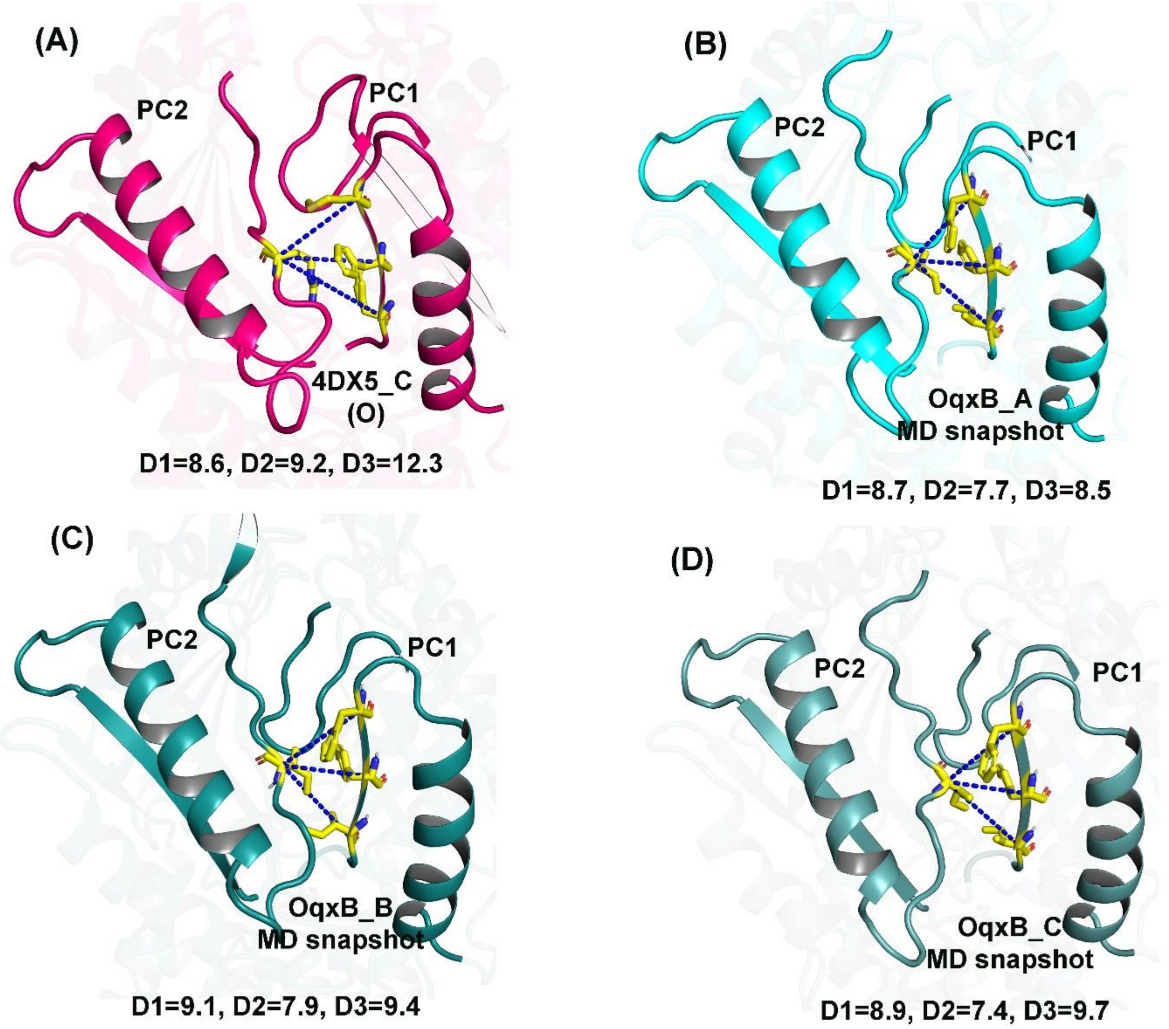

Supplementary Figure 17: Distance measurement between PC1 and PC2 sub-domain residues from MD simulation snapshots. AcrB extrusion protomer (A) distances considered as reference and MD snapshots of three protomers i.e., A, B, and C chains considered for distance measurement. The chain A (B), chain B (C) and chain C (D) distances are shown as D1 and D2 and D3 and measurement represented in Å. In *E. coli* AcrB (PDB ID: 4DX5) the D1 is between R717 and M662, D2 is between R717 and F664, D3 is R717 and F666. In *K. pneumoniae* OqxB the D1 is between I721 and F667, D2 is between I721 and F669, D3 is I721 and I671. The Cα atoms were considered for measurement.

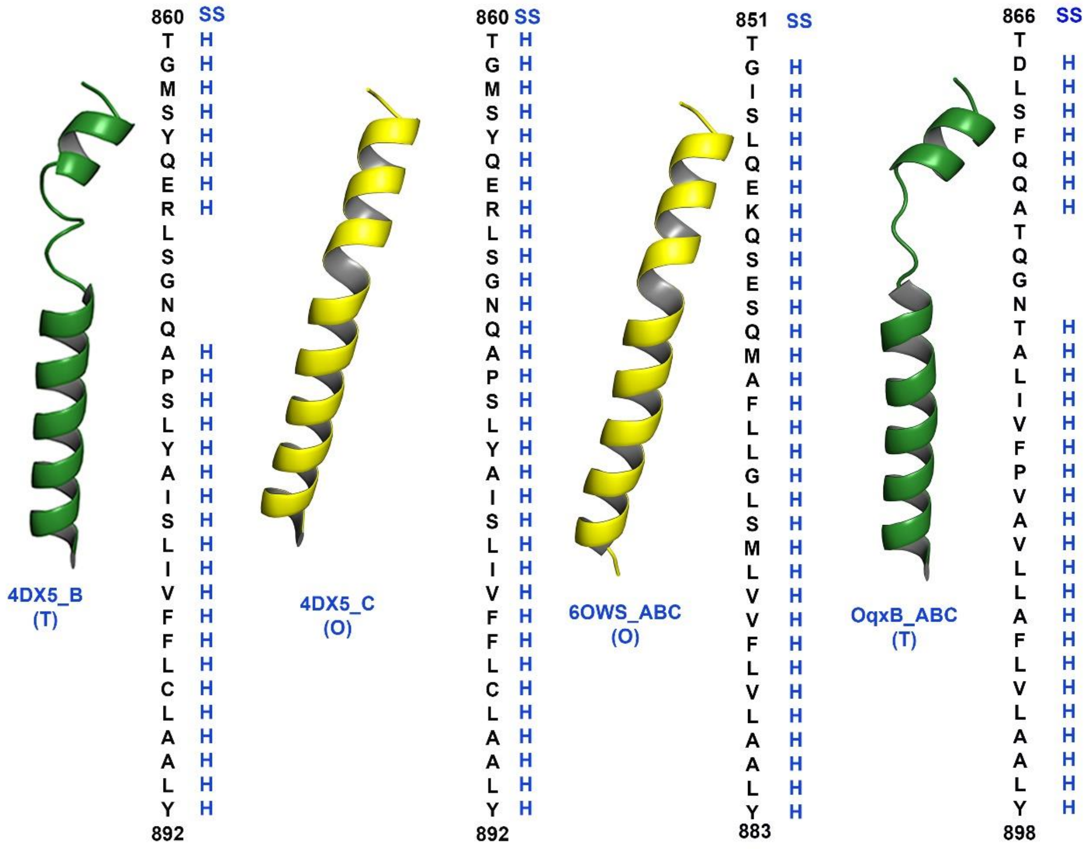

Supplementary Figure 18: TM8 secondary structural differences between tight/binding and open/extrusion protomers. The tight/binding and open/extrusion protomers from *E. coli* AcrB (PDB ID: 4DX5) considered for comparison. In *A. baumannii* AdeB (PDB ID: 6OWS) all the three protomers in open/extrusion state and in *K. pneumoniae* OqxB (PDB ID: 7CZ9) all the three protomers in tight/binding state also considered. The amino acid sequence of TM8 and secondary structural (SS) elements shown to highlight inherent extended helical nature of TM8 in open/extrusion protomers.

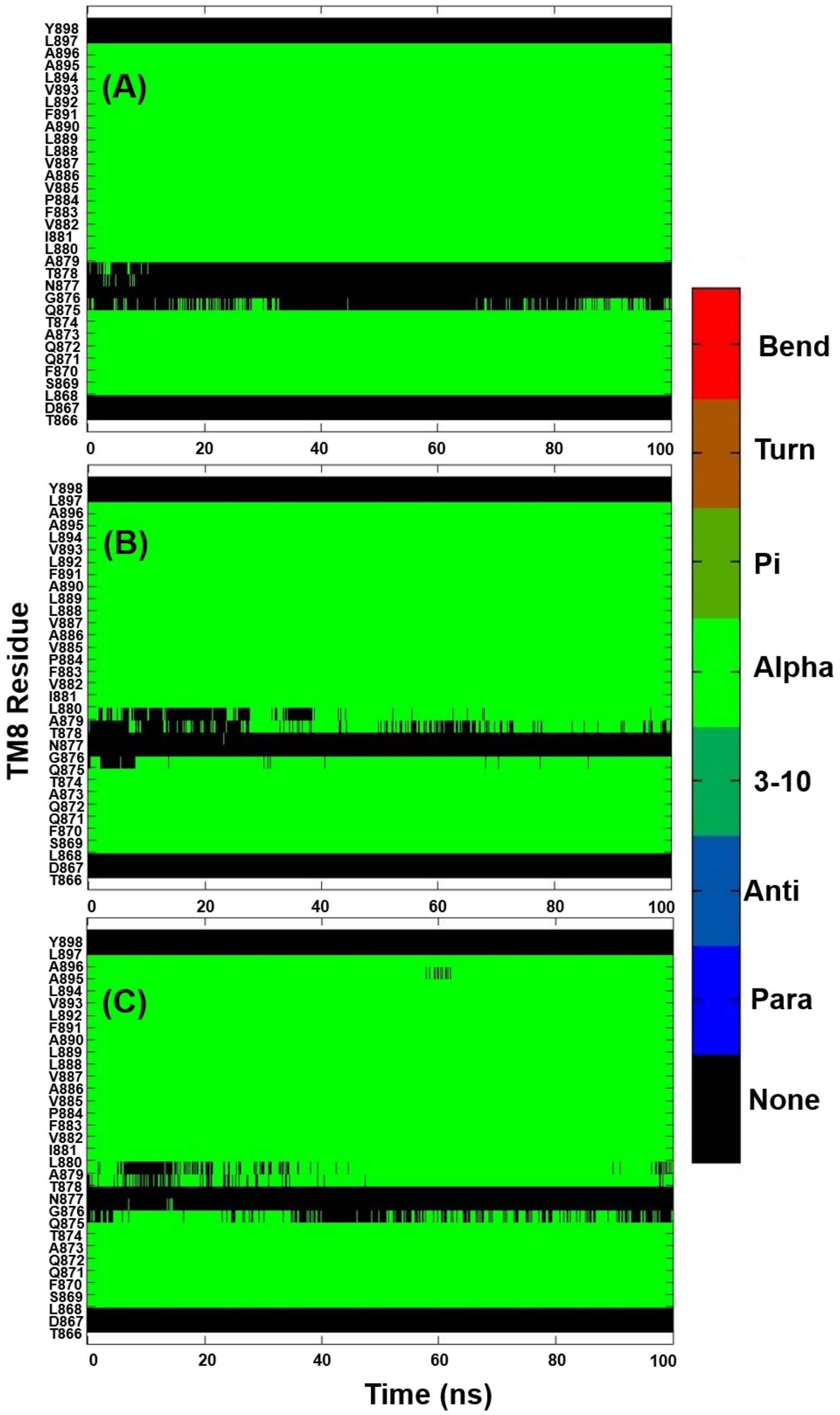

Supplementary Figure 19: Secondary structural calculations for the OqxB:ciprofloxacin complex MD simulations trajectory. The “secstruct” command implemented in Amber *cpptraj* was utilized to calculate the TM8 secondary structure elements of chain A (A), chain B (B), and chain C (C) for total simulation time.

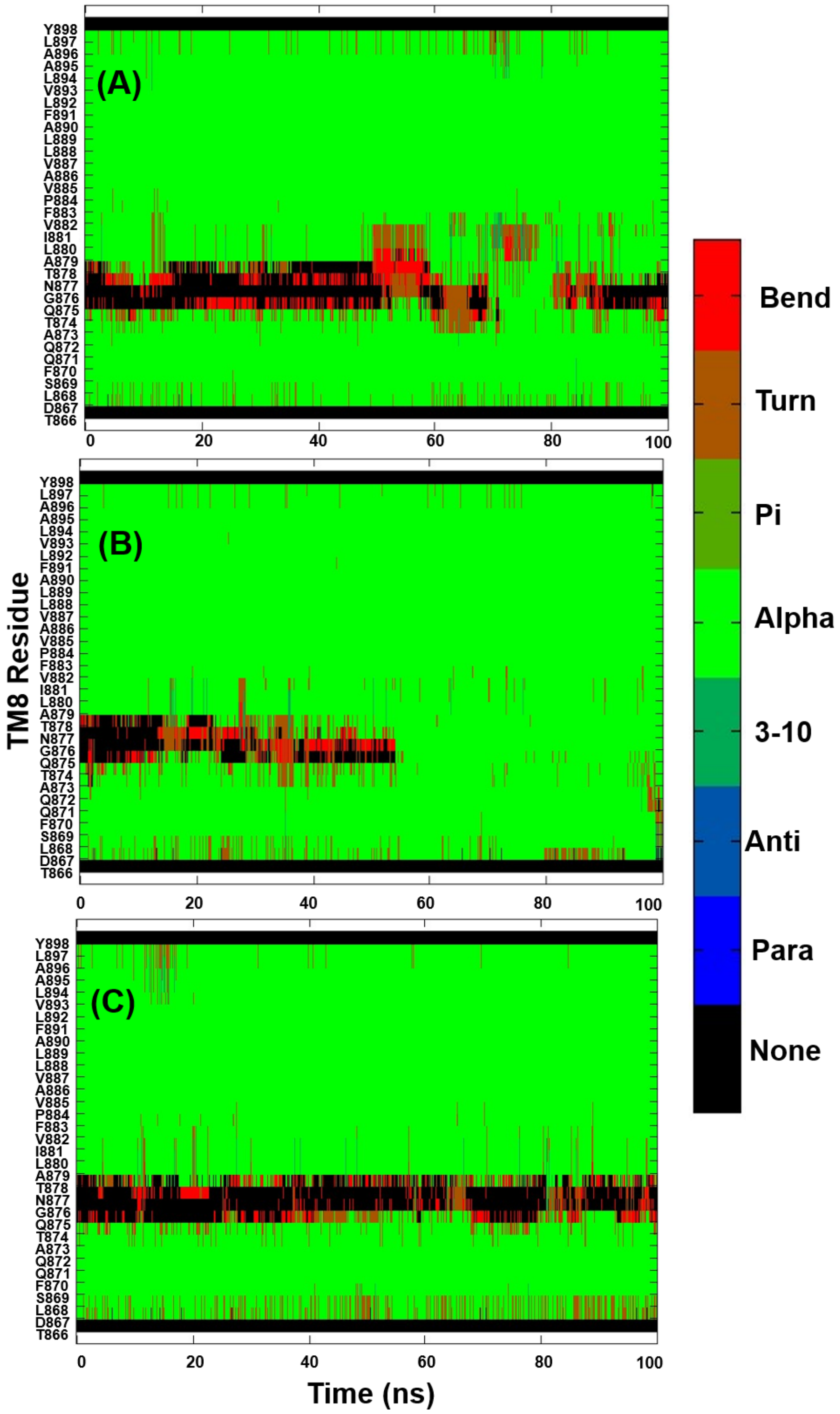

Supplementary Figure 20: Secondary structural calculations for the OqxB alone (no ligand) MD simulations trajectory. The "secstruct" command implemented in Amber *cpptraj* was utilized to calculate the TM8 secondary structure elements of chain A (A), chain B (B), and chain C (C) for total simulation time.

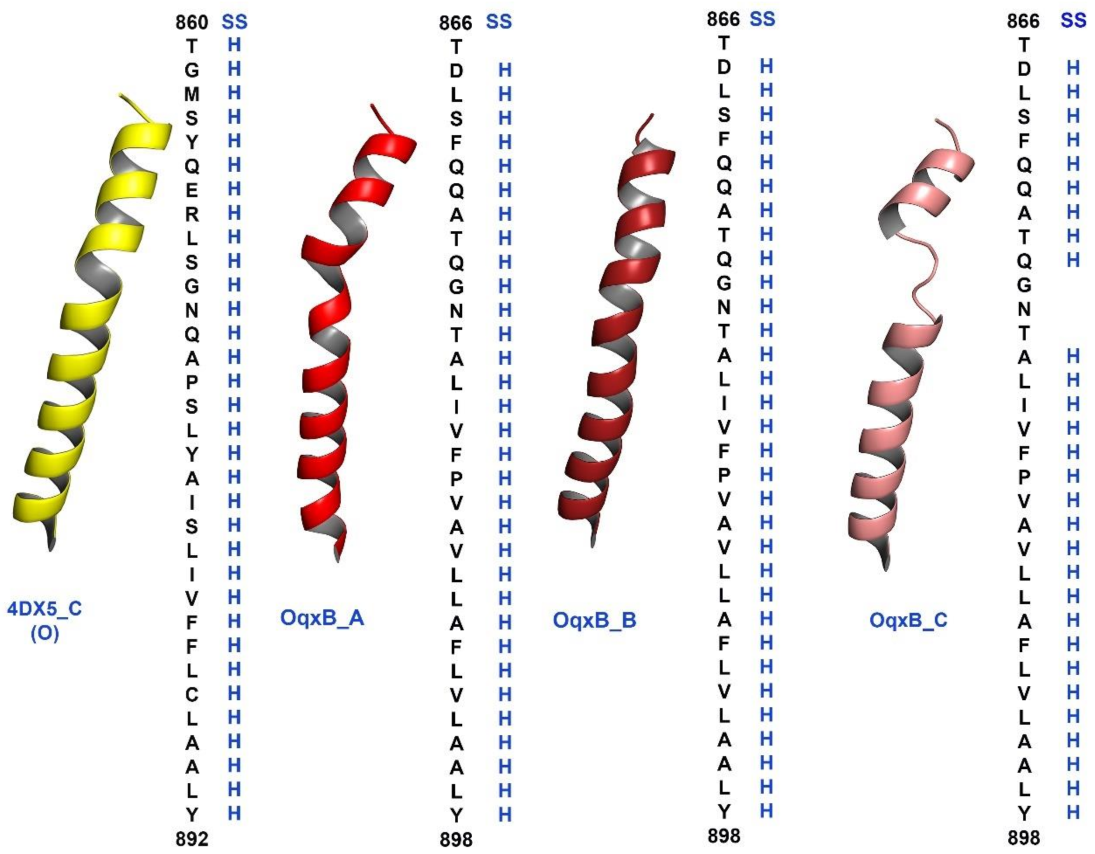

Supplementary Figure 21: TM8 secondary structural comparison between MD snapshot and AcrB open/extrusion protomer. The open/extrusion protomers from *E. coli* AcrB (PDB ID: 4DX5) considered for comparison. The amino acid sequence of TM8 and secondary structural (SS) elements shown. The A and B chains of OqxB shown extended helical nature of TM8 similar to AcrB open/extrusion protomer.

Supplementary Note: Probable resting-state determination by MD simulations.

The OqxB crystal structure exists in symmetric BBB or TTT (binding/tight) conformation. To delineate if OqxB only exists in BBB/TTT conformation or shifts to other validated conformations (access/loose or extrusion/open), accelerated molecular dynamics (aMD) simulations were performed in the absence of bound DDM molecules. The paucity of structural information of other OqxB conformations hindered using well-established tMD (targeted molecular dynamics) simulations methodology. Residues present on Cβ4 (β sheet which is present towards PC2 sub-domain) from PC1 and the short β-sheet (Cβ6) which connects PC2 with DC sub-domain are determinants of cleft movement and substrate specificity[5,6,7]. To track the cleft's opening and closing between the PC1 and PC2 domains, most of the available RND pump structures were used for distance calculations. In particular, R717 on Cβ6 (R716 in MexB and I721 in OqxB) and M662, F664, F666 on Cβ4 of AcrB (M662, F664 and F666 of MexB as well as F667, F669 and I671 of OqxB) were considered for distance measurement calculations. The Cα atom distances between individual protomer (Supplementary **Table 8**) structures is similar in access/loose (A/L), binding/tight (B/T) protomers but is >3Å less in extrusion/open (E/O) structure. This trend is consistent in all the structures irrespective of the structure determination method and the type of substrate used (Supplementary **Figure 15**). This analysis demonstrates that the cleft is open in A/L and B/T protomers whereas closed in E/O protomer. The distance calculations were carried out for MD simulations trajectories (in the

presence and absence of ligands) to understand the cleft movements (Supplementary **Figure 16**). The distances between these residues are higher in the presence of ligand and shown to be around 10 to 15 Å in all three protomers (Supplementary **Figure 16A, 16B, and 16C**). In contrast, the distance reduced to 8 to 10 Å in the absence of ligands (Supplementary **Figure 16D, 16E, and 16F**), signifying probable cleft closure. Visualizing final MD simulations snapshots of three protomers from no ligand simulations and comparing them with AcrB extrusion protomer revealed that I721 (from Cβ6 of OqxB) side chain protrudes and closes the cleft entrance by forming hydrophobic interactions with residues of Cβ4 (Supplementary **Figure 17**). The cleft closing observation by distance measurement revealed that the probable cleft closure in the absence of ligands.

The other important attribute of the E/O protomer is the extended helical nature of TM8, where a portion of TM8 possesses no secondary structure/un-structured in binding protomer (Supplementary **Figure 18**). The secondary structure prediction calculations were performed for TM8 of three chains from MD simulations trajectories to track its helical propensity. No differences were observed in the helical content from simulation trajectories performed in the presence of ligand (Supplementary **Figure 19**). In the absence of a ligand, the helical extension was observed in two out of three protomers. Helix formation was noticed between 70 and 80ns in protomer A or chain A (Supplementary **Figure 20A**). In contrast, in protomer B or chain B, it formed around 55ns simulations time and sustained throughout the remaining simulation (Supplementary **Figure 20B**). Occasional bend or turn formation was observed in protomer C or chain C (Supplementary **Figure 20C**). Comparison of TM8 helix structures of MD simulations snapshots (80th ns snapshot from chain A and 100th ns snapshot from chain B and C) with AcrB E/O protomer revealed that TM8 helix structures of chain A and B are comparable to AcrB E/O protomer (Supplementary **Figure 21**). Though we cannot detect TM8 helix extension in protomer C/chain C, cleft closure was observed from MD simulations similar to the other two protomers.

Supplementary References:

1. Seyer, K., Lessard, M., Piette, G., Lacroix, M. & Saucier, L. *Escherichia coli* Heat Shock Protein DnaK: Production and Consequences in Terms of Monitoring Cooking. *Applied and Environmental Microbiology* 69, 3231-3237 (2003).
2. Schmittgen, T. D. & Livak, K. J. Analyzing real-time PCR data by the comparative CT method. Nat. Protoc. 2008 36 3, 1101–1108 (2008).
3. Baba, T. et al. Construction of *Escherichia coli* K-12 in-frame, single-gene knockout mutants: the Keio collection. Molecular Systems Biology 2, (2006).
4. Miroux, B. and Walker, J. Over-production of Proteins in *Escherichia coli*: Mutant Hosts that Allow Synthesis of some Membrane Proteins and Globular Proteins at High Levels. Journal of Molecular Biology 260, 289-298 (1996).
5. Yu, E., Aires, J., McDermott, G. & Nikaido, H. A Periplasmic Drug-Binding Site of the AcrB Multidrug Efflux Pump: A Crystallographic and Site-Directed Mutagenesis Study. *Journal of Bacteriology* 187, 6804-6815 (2005).
6. Middlemiss, J. & Poole, K. Differential Impact of MexB Mutations on Substrate Selectivity of the MexAB-OprM Multidrug Efflux Pump of Pseudomonas aeruginosa. *Journal of Bacteriology* 186, 1258-1269 (2004).
7. Lyu, M. et al. Cryo-EM Structures of a Gonococcal Multidrug Efflux Pump Illuminate a Mechanism of Drug Recognition and Resistance. *mBio* 11, (2020).
